# Mapping DNA sequence to transcription factor binding energy *in vivo*

**DOI:** 10.1371/journal.pcbi.1006226

**Published:** 2019-02-04

**Authors:** Stephanie L. Barnes, Nathan M. Belliveau, William T. Ireland, Justin B. Kinney, Rob Phillips

**Affiliations:** 1 Division of Biology and Biological Engineering, California Institute of Technology, Pasadena, California, United States of America; 2 Department of Physics, California Institute of Technology, Pasadena, California, United States of America; 3 Simons Center for Quantitative Biology, Cold Spring Harbor Laboratory, Cold Spring Harbor, New York, United States of America; Washington University School of Medicine, UNITED STATES

## Abstract

Despite the central importance of transcriptional regulation in biology, it has proven difficult to determine the regulatory mechanisms of individual genes, let alone entire gene networks. It is particularly difficult to decipher the biophysical mechanisms of transcriptional regulation in living cells and determine the energetic properties of binding sites for transcription factors and RNA polymerase. In this work, we present a strategy for dissecting transcriptional regulatory sequences using *in vivo* methods (massively parallel reporter assays) to formulate quantitative models that map a transcription factor binding site’s DNA sequence to transcription factor-DNA binding energy. We use these models to predict the binding energies of transcription factor binding sites to within 1 *k*_*B*_*T* of their measured values. We further explore how such a sequence-energy mapping relates to the mechanisms of trancriptional regulation in various promoter contexts. Specifically, we show that our models can be used to design specific induction responses, analyze the effects of amino acid mutations on DNA sequence preference, and determine how regulatory context affects a transcription factor’s sequence specificity.

## Author summary

It has been said that we live in the “genomic era,” a time where we can readily sequence full genomes at will. However, it remains difficult to interpret much of the information within a genome. This is especially true of non-coding sequences such as promoters, which contain a number of features such as transcription factor binding sites that determine how genes are regulated. There is no straightforward regulatory “code” that tells us how transcription factor binding sites are organized within a promoter. In this work we examine how DNA sequence determines one of the most important features of a promoter, the strength with which a transcription factor binds to its DNA binding site. We discuss an approach to modeling DNA sequence-specific transcription factor binding energies *in vivo* using a massively parellel reporter assay. We develop models that allow us to predict the binding energy between a transcription factor and a mutated version of its binding site. We then show that this modeling technique can be used to address a number of scientific and design questions, such as engineering the behavior of genetic circuit elements or examining how transcription factors and their binding sites co-evolve.

## Introduction

High-throughput sequencing allows us to sequence the genome of nearly any species at will. The amount of genomic data available is already enormous and will only continue to grow. However, this mass of data is largely uninformative without appropriate methods of analyzing it. Despite decades of research, much genomic data still defies our efforts to interpret it. It is particularly challenging to interpret non-coding DNA such as intergenic regulatory regions. We can infer the locations of some transcription start sites and transcription factor binding sites, but these inferences tell us little about the functional role of these putative sites. In order to better interpret these types of sequences, we need a better understanding of how sequence elements control gene expression. A deep understanding of the relationship between DNA sequence and gene expression would enable one to a) predict the binding strengths of novel transcription factor binding sites and b) design regulatory sequences *de novo* for synthetic biology applications. An important avenue for developing this level of understanding is to propose models that map sequence to function and to perform experiments that test these models.

One challenge that has made it difficult to develop quantitative sequence-function mappings is the fact that an extremely small portion of known regulatory sequences are understood on a quantitative biophysical level. Over half of the genes in *Escherichia coli*, which is arguably the best-understood model organism, lack any regulatory annotation (see RegulonDB [1]). Those operons whose regulation is well described (e.g. the *lac*, *rel*, and *mar* operons [2–4]) required decades of study involving laborious genetic and biochemical experiments [5]. A wide variety of new techniques have been proposed and implemented to simplify the process of determining how a gene is regulated. Chromatin immunoprecipitation (ChIP) based methods such as ChIP-chip and ChIP-seq make it possible to determine the genome-wide binding locations of individual transcription factors of interest. Massively parallel reporter assays (MPRAs) have made it possible to read out transcription factor binding position and occupancy *in vivo* with base-pair resolution, and provide a means for analyzing additional features such as “insulator” sequences [6–8]. *In vitro* methods based on protein-binding microarrays [9], SELEX [10–12], MITOMI [13–15], and binding assays performed in high-throughput sequencing flow cells [16, 17] have made it possible to measure transcription factor affinity to a broad array of possible binding sites and can also account for features such as flanking sequences [15, 18, 19]. However, *in vitro* methods cannot fully account for the *in vivo* consequences of binding site context and interactions with other proteins. Current *in vivo* methods for measuring transcription factor binding affinities, such as bacterial one-hybrid [20, 21], require a restructuring of the promoter so that it no longer resembles its genomic counterpart. Additionally, efforts to computationally ascertain the locations of transcription factor binding sites frequently produce false positives [22, 23]. Furthermore, a common assumption underlying many of these methods is that transcription factor occupancy in the vicinity of a promoter implies regulation, but it has been shown that occupancy cannot always accurately predict the effect of a transcription factor on gene regulation [24, 25]. As these examples show, it remains challenging to integrate multiple aspects of transcription factor binding into a cohesive understanding of gene regulation that would allow for predictive models that map sequence to function.

In previous work we showed how an MPRA called Sort-Seq can be used on virgin promoters to identify regulatory architectures [26]. The current work takes the logical and critical next step of rigorously examining how reliable the Sort-Seq results are as a foundation for predicting and controlling transcription with single-nucleotide resolution. In Ref. [27], we showed that the MPRA Sort-Seq [28], combined with a simple linear model for protein-DNA binding specificity, can be used to accurately predict the binding energies of multiple RNAP binding site mutants, serving as a jumping off point for the use of such models as a quantitative tool in synthetic biology. Here we apply this technique to transcription factor binding sites in an effort to better understand how transcription factors interact with regulatory DNA under different conditions. Specifically, we use Sort-Seq to map sequence to binding energy for a repressor-operator interaction, and we rigorously characterize the variables that must be considered in order to obtain an accurate mapping between DNA sequence and binding energy. We then use our sequence-energy mapping to design a series of operators with a hierarchy of controlled binding energies measured in absolute energy units (*k*_*B*_*T*). To demonstrate our control over these operators and their associated regulatory logic, we use these characterized binding sites to design a wide range of induction responses with different phenotypic properties such as leakiness, dynamic range and [*EC*_50_]. Next, we focus our attention on the synergy between mutations in the amino acid sequence of transcription factors and their corresponding binding sites. Finally, we show the broader reach of these results by exploring how binding site position and regulatory context can change the DNA-protein sequence specificity for multiple different transcription factors.

## Results

### Obtaining energy matrices using Sort-Seq

A major goal of this study was to show that one can use Sort-Seq to precisely map DNA sequence to binding energy for a transcription factor binding site, thus making it possible to predict and manipulate transcriptional activity *in vivo*. While numerous *in vitro* studies have successfully mapped sequence to affinity [9–17, 29, 30], and some *in vivo* studies have used methods such as bacterial one-hybrid to provide such mappings as well [20, 21], these studies are limited because they do not reflect the actual wild-type arrangement of regulatory elements, thus potentially missing vital regulatory information. Moreover, while position-weight matrices (PWMs) derived from genomic data have traditionally been used to ascertain *in vivo* sequence specificities, it can be difficult to convert these specificities into quantitative binding energy mappings due to the relatively small number of sequences that are used to generate these PWMs.

**Fig 1:**
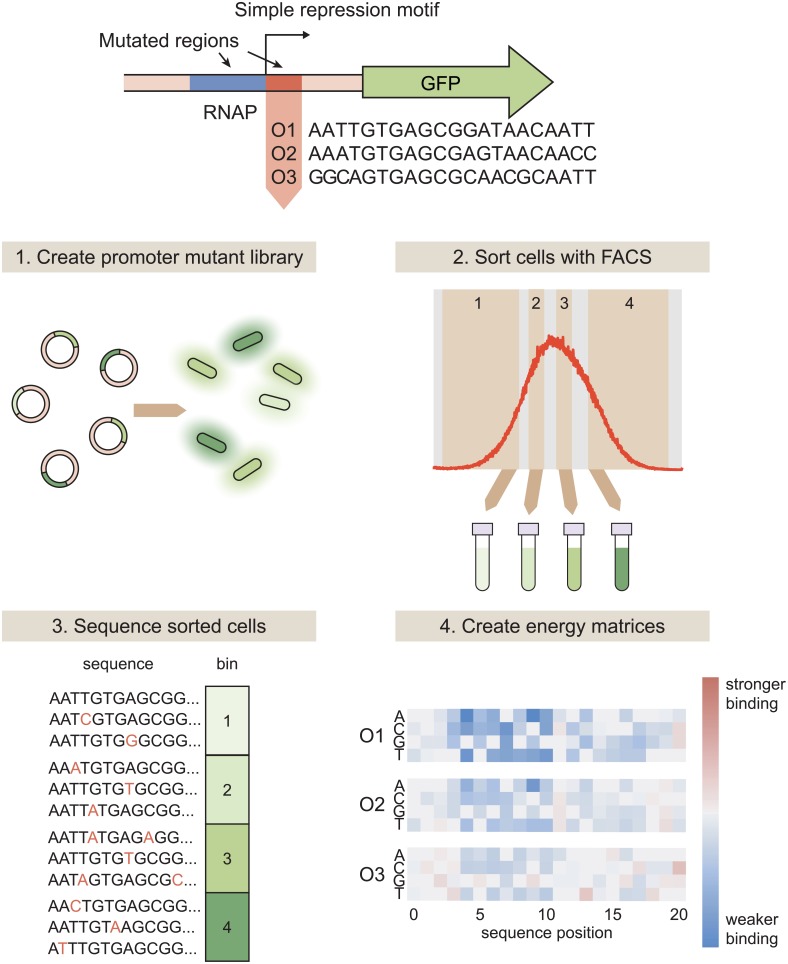
Using Sort-Seq to obtain energy matrices. To begin, we design a simple repression motif in which a repressor binding site is placed immediately downstream of the RNAP site. When RNAP binds, it initiates transcription of the GFP reporter gene. We analyze simple repression constructs using each of the three natural *lac* operators, O1, O2, and O3. Sort-Seq then proceeds as follows. 1. We create a mutant library in which the RNAP and operator sequences are randomly mutated at a rate of approximately 10%, and transform this library into a cell population such that each cell contains a different mutant operator sequence. 2. To measure gene expression, we sort the cell population into bins based on fluorescence level. 3. We then sequence variant promoter sequences within each bin. The bin in which each promoter is found serves as a measure of that promoter’s activity. 4. From this information, we can infer an energy matrix for the repressor binding site indicating which mutations result in a higher or lower binding energy relative to the reference sequence. Energy matrices can either be inferred using all of the Sort-Seq data, or the Sort-Seq data can be split into multiple “replicates” to obtain replicate energy matrices that can be used to estimate error.

Sort-Seq has previously been shown to be a promising technique for mapping protein binding sequences to binding energies. In Ref. [27], binding energy predictions for RNAP were made from an energy matrix generated in Ref. [28] that used the wild-type *lac* promoter as a reference sequence (i.e. the sequence that was mutated to perform Sort-Seq). Here, we design experiments that use the Sort-Seq technique described in [28] with the specific intent of creating energy matrices with maximum predictive power (see Fig 1), and we test the predictions from these matrices against measured binding energies. We show that such predictive matrices can be produced for multiple transcription factors (e.g. XylR, PurR, and LacI) implicated in an array of regulatory architectures. To thoroughly test the accuracy of our predictive matrices, we begin with promoters that employ “simple repression,” in which a repressor binds to an operator such that it occludes RNAP binding, thereby preventing transcription and repressing the gene [31]. As a model for how sequence-energy mappings might be used for transcription factor binding sites in simple repression architectures, we interrogate the binding specificity of the *lac* repressor (LacI). LacI was chosen for this role because it is well-characterized and has known binding sites in only one operon within the genome, making it an ideal choice for this kind of systematic and rigorous analysis. We create three distinct energy matrices in which each of the natural *lac* operators (O1, O2, or O3 [2]) acts as the reference sequence. S1 Fig lists the wild-type sequences for these simple repression constructs.

As described in Fig 1, to perform Sort-Seq we start by mutating the promoter at a rate of ∼ 10%. Here we mutate both the RNAP binding site and the operator, starting with either O1, O2, or O3 for the operator sequence. While our analysis focuses on the operators themselves, mutating the RNAP site as well aids in model-fitting as described in S1 Text. We place the promoters upstream of a fluorescent reporter gene and create a plasmid library of these constructs. We transform this plasmid library into a population of *E. coli* in which *lacI* and *lacZYA* have been deleted, but *lacI* has been reintroduced to the genome with a synthetic RBS that allows us to precisely control the LacI copy number within the cell, as described in Ref. [32]. We require at least 10^6^ transformants for each plasmid library to ensure sufficient library diversity. Then, we use fluorescence-activated cell sorting (FACS) to sort *E. coli* containing these plasmids into four bins based on their expression levels. We perform high-throughput sequencing on the libraries from each bin. For the majority of our analysis, we split the sequencing results into three separate “replicates.” As discussed in S1 Text, this provides a level of variation that is comparable to multi-day biological replicates.

Once we have a set of promoter sequences with corresponding expression levels, we use these data to infer an energy matrix for the transcription factor binding site that assigns energy values (in arbitrary units) to each base at each sequence position. We use Markov Chain Monte Carlo (MCMC) with a Metropolis-Hastings algorithm to infer a set of energy matrix values that maximizes the mutual information between the predicted binding energies of our sequences and their corresponding expression bins. Briefly, this algorithm proceeds as follows: 1) a set of energy matrix parameters is proposed for each base at each operator position, 2) the proposed matrix is used to calculate the binding energies (in arbitrary units) of the set of binding site sequences, and 3) this set of energy parameters is accepted or rejected with some probability. If it is rejected, a new set of energy parameters is proposed, some distance away from the previous parameter set. As discussed in detail in Ref. [33], the probability of accepting a proposal is derived from the probability distribution *p*(data|model) ∝ 2^*NI*(*ε*(*σ*);*μ*)^, where *N* is the number of data points and *I*(*ε*(*σ*); *μ*) represents the mutual information between the energy prediction *ε*(*σ*) for the promoter sequence *σ* and the expression bin *μ*. In other words, the set of proposed values is accepted or rejected according to how well the associated binding energy predictions explain the observed distribution of sequences in expression bins. After this algorithm has been iterated 30000 times (after 10000 repeats for a “burn-in” period), the energy matrix values are then determined by calculating the sample mean of the parameter values that were accepted. Once an energy matrix has been inferred, the matrix is fixed such that the matrix elements corresponding to the reference sequence are set at 0, and the other matrix elements at each sequence position are calculated relative to this reference element. The reference sequence is the operator sequence that serves as the “wild-type” in each Sort-Seq experiment–that is, the sequence away from which the library sequences are mutated. For more details on this procedure, see S1 Text.

The energy matrices that result from the procedure described above are given in arbitrary energy units. To convert these arbitrary units into absolute energy units, we also perform Bayesian parameter estimation using MCMC to determine the scaling factor that should be applied to the energy matrix to convert each position into *k*_*B*_*T* energy units (we note that 1 *k*_*B*_*T* ≈ 0.62 kcal/mol at *T* = 37°C). This inference procedure is highly similar to the procedure outlined above, but where before we maximized the mutual information between predicted binding energies and expression bin for a full set of energy matrix parameters, here we maximize the mutual information between predicted expression and expression bin for a single parameter, which is the scaling factor that converts a matrix into absolute energy units. This scaling factor is a “diffeomorphic mode” of the model that cannot be inferred by direct mutual information maximization, but can be inferred when incorporated into a more complex model in which other sequence elements are also varied [34]. We make use of the assumption that gene expression will be proportional to the probability that RNAP is bound to the promoter, *p*_*bound*_, which is given by

$$\begin{array}{c} p_{bound}=\frac{\frac{P}{N_{NS}}e^{-\beta \Delta \varepsilon_{P}}}{1+\frac{P}{N_{NS}}e^{-\beta \Delta \varepsilon_{P}}+\frac{2R}{N_{NS}}e^{-\beta \Delta \varepsilon_{R}}}, \end{array}$$ (1)

where *P* is the number of RNAP in the cell, Δ*ε*_*P*_ is the RNAP binding energy, *R* is the repressor copy number, *N*_*NS*_ is the number of nonspecific binding sites in the genome, and Δ*ε*_*R*_ is the repressor binding energy. As noted in S1 Text, our inference procedure also requires that we infer an energy matrix and scaling factor for the RNAP binding site. We do not note these values in this work, as our focus is on sequence-energy mappings for transcription factor binding sites. We note also that we can write the repressor binding energy as Δ*ε*_*R*_ = *αε*_mat_ + Δ*ε*_wt_, where *ε*_mat_ is the energy value obtained by summing the matrix elements associated with a sequence, Δ*ε*_wt_ is the binding energy associated with the reference sequence, and *α* is the desired scaling factor that converts the matrix values into *k*_*B*_*T* units. To obtain a value for *α*, an MCMC algorithm is used where we maximize the mutual information *I*(*p*_*bound*_, *μ*) between the values of *p*_*bound*_ calculated for each binding site sequence and the expression bin into which that sequence was sorted. For more information on this process, see S1 Text. See S2 Text for a comparison to other methods for obtaining the scaling factor.

### Choice of reference sequence can alter the repressor’s apparent sequence specificity

A reference sequence refers to the sequence which serves as the “wild-type” for each experiment. For each library, the promoter is mutated relative to its reference sequence. Additionally, when assigning binding energies to an energy matrix, all binding energies are calculated relative to the reference sequence. One might assume that Sort-Seq experiments should reveal the same binding specificity regardless of the reference sequence used to produce the library, provided that the transcription factor does not change. To test this possibility, we generated energy matrices using three different reference sequences, all of which are binding sites for LacI. For our reference sequences we use the three natural *E. coli* *lac* operators (O1 = AATTGTGAGCGGATAACAATT, O2 = AAATGTGAGCGAGTAACAACC, and O3 = GGCAGTGAGCGCAACGCAATT).

For our primary analysis we use single-point energy matrix models. These models assume that each nucleotide position within a binding site contributes independently to the binding energy (see S3 Text for predictions using higher-order models). Each operator has a distinct LacI binding energy, with O1 being the strongest at -15.3 *k*_*B*_*T*, O2 being the second strongest at -13.9 *k*_*B*_*T*, and O3 being the weakest at -9.7 *k*_*B*_*T* [32]. The operator sequences are rather dissimilar to each other, with O2 having 5 mutations relative to O1 and O3 having 8 mutations relative to O1 (and 11 mutations relative to O2). For each library, the average operator sequence has only 2 mutations relative to the reference sequence. As a result, a library generated with O1 as the reference sequence is unlikely to share any mutant sequences with a library generated with O2 or O3 as the reference sequence. Here we assess whether dissimilar mutant libraries generated from different reference sequences produce similar energy matrices and sequence logos from their respective Sort-Seq data sets.

**Fig 2:**
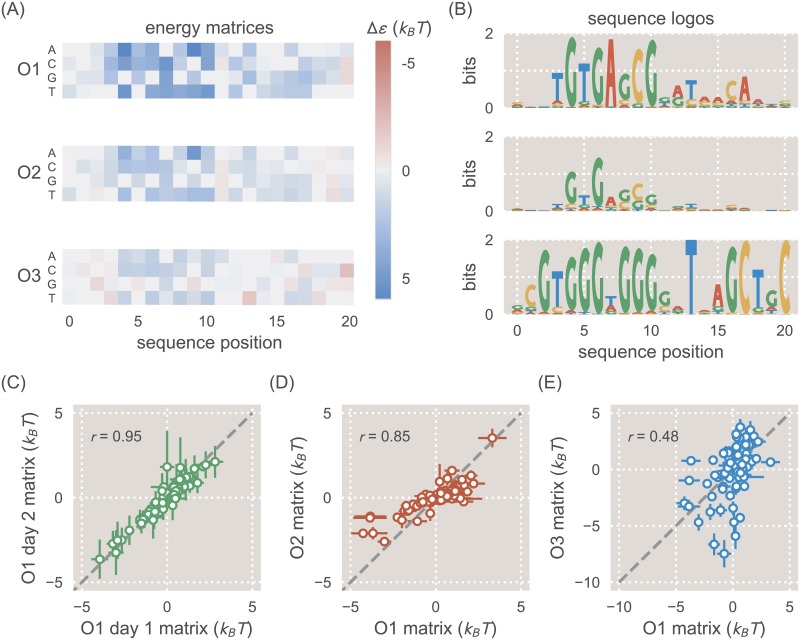
Energy matrices and sequence logos for the natural *lac* operators. A: Energy matrices show how mutations can be expected to affect binding energy. Reference sequences for each energy matrix (either the O1, O2, or O3 sequence) have been set at 0 *k*_*B*_*T* (gray squares), and the energy values at all other positions of the matrix are thus relative to the reference sequence. Red squares represent mutations that create a stronger binding energy than the reference sequence, and blue squares represent mutations that create a weaker binding energy. In columns where multiple squares are gray, this indicates that there is no significant change in binding energy relative to the reference sequence. B: While the energy matrices are qualitatively similar for all three operators, the sequence logos indicate clear differences in the information that can be provided by each operator. The O1 and O2 operators produce similar sequence logos, but the O3 sequence logo incorrectly predicts the preferred binding sequence for LacI. The O3 sequence logo also indicates a much lower information content than for O1 and O2. C: Two separate biological replicates of a matrix derived from the O1 reference sequence (with repressor copy number *R* = 62) are plotted against one another. D: The O1 energy matrix is plotted against the O2 energy matrix, both derived from strains with *R* = 130. E: The O1 energy matrix is plotted against the O3 energy matrix, both derived from strains with *R* = 130.

We obtain energy matrix models by following the Sort-Seq procedure outlined above, splitting our Sort-Seq data into three separate groups of nonoverlapping sequences to produce matrix replicates, as discussed in S1 Text. We then infer an energy matrix for each replicate. In Fig 2 we show energy matrices composed of the mean energy value at each matrix position, and the corresponding sequence logos. As shown in Fig 2A, the three operators each produce qualitatively similar energy matrices, with the left side of the binding site showing greater sequence dependence than the right side, as evidenced by the larger magnitude of the binding energies assigned to each matrix position. Note that we set the binding energy of the reference sequence to 0 *k*_*B*_*T* for these energy matrices, so that the binding energies assigned to each possible mutation are calculated relative to the reference sequence. For all energy matrices, positions 4-10 show the greatest sequence preference. This preference is reflected in the natural *lac* operator sequences themselves, as the bases from 4-10 are conserved in each of the operators. Notably, the majority of mutations available to O1 incur a penalty to binding energy, while many of the mutations available to O3 enhance the binding energy. This is consistent with the observation that O1 has a strong binding energy while O3 has a weak binding energy.

When the energy matrices are used to produce sequence logos as in Fig 2B (see Ref. [35] for an explanation of the mathematics used to relate binding energies to base-pair frequencies, and Ref. [36] for a discussion of sequence logos themselves), we see a consistent preference for a slightly asymmetric binding site, reflecting the fact that LacI is known to bind asymmetrically to its operators [37]. Additionally, clear differences arise for the different operators. While the sequence logos derived from O1 and O2 indicate very similar sequence preferences, the preferred sequence suggested by the O3 sequence logo differs in some prominent positions. In S4 Text we note that weaker binding sites exhibit a greater variation in the quality of their sequence logos; thus it may be that the O3 binding site is simply too weak to provide an informative sequence logo.

In Fig 2C–2E we plot the energy values from each matrix against one another to show how the energy matrices compare to one another quantitatively. For ease of comparison, here the energy values are fixed so that the mean energy value at each sequence position is 0 *k*_*B*_*T*. We see that replicates of energy matrices with an O1 reference sequence are highly similar to each other with a Pearson’s correlation coefficient of *r* = 0.95, as calculated from the mean energy matrix values (Fig 2C). However, this similarity deteriorates somewhat as the reference sequence diverges from O1, with a value of *r* = 0.85 for an O2-derived matrix (Fig 2D) and *r* = 0.48 for an O3-derived matrix (Fig 2E). Thus, while energy matrices derived from different reference sequences may be qualitatively similar, there are notable quantitative dissimilarities between these matrices.

In S2 Fig we also represent each matrix as an energy logo as introduced in Ref. [38] and used in Ref. [30] to represent the sequence specificity of LacI determined from *in vitro* experiments. In S3 Fig we show how energy matrix values inferred for O1 in this work compare with the energy matrix values inferred for O1 using an *in vitro* technique in Ref. [30]. We find that the values from these two studies generally agree, particularly for lower energy values. Any differences may be due to the different experimental procedures used in these studies.

### Energy matrix models predict measured energy values

The energy matrices obtained via Sort-Seq should allow us to map sequence to phenotype. The relevant phenotype for simple repression constructs is the degree to which the system is repressed, which can be measured using the fold-change. We define fold-change as the ratio of expression in a repressed system to expression in a system with no repressors, as described by the equation

$$\begin{array}{c} \text{fold-change}=\frac{\text{expression}(R)}{\text{expression}(R=0)}. \end{array}$$ (2)

where *R* is the repressor copy number. As discussed in further detail elsewhere [31, 32], the fold-change can also be computed using a thermodynamic model given by

$$\begin{array}{c} \text{fold-change}=\frac{1}{1+\frac{2R}{N_{NS}}e^{-\beta \Delta \varepsilon_{R}}}, \end{array}$$ (3)

where the factor of 2 in “2*R*” indicates that for the case of LacI, each LacI tetramer has two heads and can essentially be counted as two repressors. *N*_*NS*_ is the number of nonspecific binding sites available in the genome (∼ 4.6 × 10^6^ in *E. coli*) and Δ*ε*_*R*_ is the operator binding energy. We note that this model makes the simplifying assumption that the RNAP binds weakly to the promoter. We find that for the *lacUV5* promoter, this assumption holds for RNAP copy number *P* ≲ 1000. From Ref. [39] we know that the relevant sigma factor, RpoD, has a copy number of 650 ± 100 in the growth condition used here (M9 + 0.5% glucose at 37°C).

In principle, the energy matrix models shown in Fig 2 can be used to predict the binding energy of an operator mutant. To explore the ability of energy matrices to predict the effects of mutations on operator binding strength, we designed a number of mutant operators with 1, 2, or 3 mutations relative to the O1 operator. Experimentally-determined values for the binding energies of these mutants could then be compared against values predicted by our LacI energy matrices.

**Fig 3:**
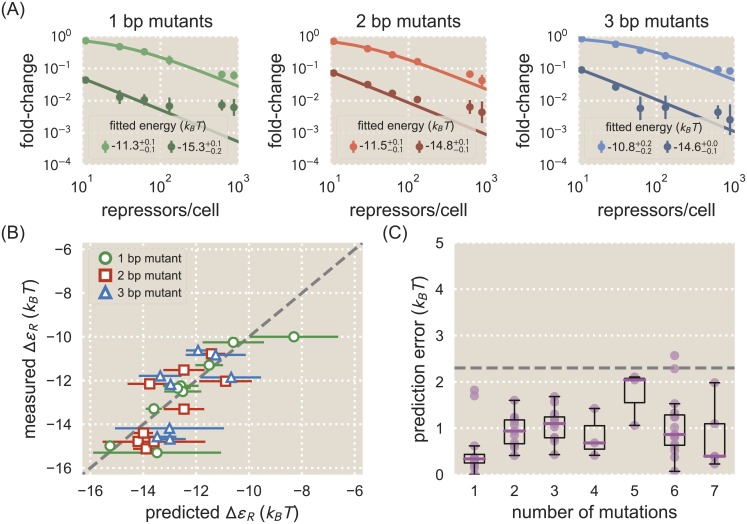
Energy matrix predictions compared to binding energies derived from fold-change data. A: Fold-change data were obtained by flow cytometry for each of the mutant operators by measuring their respective fluorescence levels at multiple LacI copy numbers and normalizing by the fluorescence when *R* = 0. The solid lines in each plot represent a fold-change curve that has been fitted to the data set to obtain a binding energy measurement. Each plot shows data and fits for two operator mutants, one weak and one strong, for 1 bp (left), 2 bp (middle), and 3 bp (right) mutants. The fitted energy values are shown for each mutant, where the superscripts and subscripts represent the 95% confidence interval for the fit. All remaining data is shown in S2–S4 Figs. Approximately 30 operator mutants were measured in total. We note that lower expression measurements are less accurate than higher expression measurements due to autofluorescence and limitations in the flow cytometer’s ability to measure weak signals. This adversely affects the accuracy of fold-change values for strongly repressed strains. B: The measured binding energy values Δ*ε*_*R*_ (y axis) are plotted against binding energy values predicted from an energy matrix derived from the O1 operator (x axis). The horizontal error bars represent the standard deviation of predictions made from three matrix replicates obtained by splitting the Sort-Seq data into three groups. MCMC was used to obtain a scaling factor for each matrix to convert it into *k*_*B*_*T* units. The vertical error bars represent the 95% confidence interval of the fitted Δ*ε*_*R*_ values (where not visible, these error bars are smaller than the marker). While the quality of the binding energy predictions does appear to degrade as the number of mutations relative to O1 is increased, the O1 energy matrix is still able to approximately predict the measured values. C: Binding energies for each mutant were predicted using both the O1 and O2 energy matrices and compared against measured binding energy values. The prediction error, defined as the magnitude of the difference in *k*_*B*_*T* between a predicted binding energy and the corresponding measured binding energy, is plotted here against the number of mutations relative to the reference sequence whose energy matrix was used to make the prediction. Each data point is shown in purple, and box plots representing the data are overlaid to clearly show the median error and variability in error. For sequences with 4 or fewer mutations, the median prediction error is consistently lower than 1.5 *k*_*B*_*T*. The dashed horizontal line represents the point at which the error corresponds to an approximately 10-fold difference in fold-change.

To obtain experimental values for mutant binding energies we start with chromosomally-integrated simple repression constructs for each mutant, which were incorporated into strains with LacI tetramer copy numbers of *R* = 11 ± 1, 30 ± 10, 62 ± 15, 130 ± 20, 610 ± 80, and 870 ± 170. These copy numbers were inferred from quantitative Western blot measurements in Ref. [32], and the error in these copy numbers denotes the standard deviation of at least three Western blot replicates. We determined the fold-change by measuring the YFP fluorescence levels of each strain by flow cytometry and substituting them into Eq 2. We determine each mutant’s binding energy, Δ*ε*_*R*_, by performing a single-parameter fit of Eq 3 to the resulting data via nonlinear regression. Fig 3A shows several fold-change values for 1 bp, 2 bp, and 3 bp mutants overlaid with these fitted curves (the remaining fold-change data are shown in S2–S4 Figs).

The energy matrices derived from Sort-Seq can be used to predict the value of Δ*ε*_*R*_ associated with a given operator mutant, as discussed in detail in S1 Text. To make these predictions, we use energy matrices produced using Sort-Seq data where O1 is the reference sequence and repressor copy number *R* = 130. As before, we split the Sort-Seq data into three groups to produce three replicate energy matrices. We make our predictions using each of these replicate matrices in order to obtain a mean value and standard deviation for the predicted Δ*ε*_*R*_ for each operator mutant. We note that any error in the predictions can be caused by error in the energy matrices themselves or in the inferred scaling factors. Fig 3B shows how binding energy values measured by fitting to repressor titration data compare to values predicted using energy matrices that were produced using O1 as a reference sequence. For single base pair mutations most predictions perform well and are accurate to within 1 *k*_*B*_*T*, with many predictions differing from the measured values by less than 0.5 *k*_*B*_*T*. Predictions are less accurate for 2 bp or 3 bp mutations, although the majority of these predictions are still within 1.5 *k*_*B*_*T* of the measured value. To give a sense of the consequences of an incorrect energy prediction, a prediction error of ±1 *k*_*B*_*T* can alter the expected fold-change of a simple repression architecture by a magnitude of approximately 0 − 0.25, depending on the binding site’s binding energy and the repressor copy number *R*.

The quality of matrix predictions appears to degrade as mutants deviate farther from the wild-type sequence used to generate the energy matrix. To evaluate predictions for a broader range of deviations from the energy matrix, we made predictions from two energy matrices: the mean energy matrix using O1 as a reference sequence with repressor copy number *R* = 130, and the mean energy matrix using O2 as a reference sequence with *R* = 130. This allowed us to access predictions for operators that are mutated by several base pairs relative to the matrix. In Fig 3C we show how prediction error, defined as the discrepancy in *k*_*B*_*T* between a predicted and measured energy value, varies depending on the number of mutations relative to the wild-type binding site sequence. We find that predictions remain relatively accurate for mutants that differ by up to 4 bp relative to the wild-type sequence, with median deviations of ∼ 1.0 *k*_*B*_*T* or less from the measured binding energy. Other studies have noted that energy matrix models that don’t account for epistatic interactions fail to accurately predict binding energies for mutants with multiple mutations relative to the reference sequence [29, 40]. Thus we find that the relatively low errors depicted in Fig 3C exceed expectations for what a single-point energy matrix model can achieve.

We note that energy matrix quality, as measured by the accuracy of its predictions, may be affected by factors such as repressor copy number or wild-type transcription factor binding energy. Changes in growth state can be expected to affect the number of transcription factors present in the cell (although transcription factors appear to be less sensitive to growth state than the general proteome [39]), so it is important to determine how sensitive our approach is to changes in repressor copy number. Additionally, we expect that at some repressor copy numbers and binding site strengths, the binding site may be either fully saturated with repressor or entirely unbound by repressor, which may also affect energy matrix quality. In S4 Text, we assess whether energy matrix quality is affected by the LacI copy number of the background strain, and find that it has little effect on matrix quality. We also compare predictions made from energy matrices with different reference sequences (i.e. O1, O2, or O3), and find that using O1 as a reference sequence produces the most accurate energy matrices, while using O3 produces energy matrices that are almost entirely non-predictive. In S5 Text, we consider whether better energy matrices are made using libraries in which the entire promoter is mutated or only the operator is mutated. We find that mutating the operator alone can provide more accurate energy matrices, though one must fit energy matrix predictions to binding energy measurements in order to convert these matrices into *k*_*B*_*T* units.

### Designed induction responses

Our predictive energy matrices suggest a promising strategy for addressing the challenge of genetic circuit design, which has typically relied on trial and error to achieve specific outputs [41, 42]. By contrast, previous studies have shown how thermodynamic models can be used to predict gene outputs given a set of inputs [31, 32], which can suggest appropriate inputs to produce a desired output. For example, the key inputs for the fold-change Eq 3 are repressor copy number *R* and repressor-operator binding energy Δ*ε*_*R*_, and one can use Eq 3 to determine a set of *R* and Δ*ε*_*R*_ values that can be used to target a desired fold-change response. Energy matrix predictions can be used to design operator sequences with a particular value of Δ*ε*_*R*_, thereby making it possible to tune genetic circuits and target specific phenotypes. As shown in Fig 3B, mutating an operator by as little as one base pair can provide a broad range of Δ*ε*_*R*_ values that can be predicted accurately.

One particularly useful class of simple genetic circuit, which can be layered with other genetic components to create complex logic [43], is inducible simple repression [44–47]. In such a system, an allosteric repressor can switch between an active form, which binds to an operator with high affinity, and an inactive form, which has a low affinity to the operator. An inducer may bind to the repressor and stabilize the repressor’s inactive form, thereby reducing the probability that the repressor will bind to the operator and increasing the probability that RNAP will bind and initiate transcription. The result is that an inducible system can access a broad range of fold-change values simply by tuning the concentration of inducer. As discussed in Ref. [48], the fold-change of an inducible simple repression circuit can be described by the equation

$$\begin{array}{c} \text{fold-change}\left(c\right)=(1+\frac{{\left(1+\frac{c}{K_{A}}\right)}^{n}}{{\left(1+\frac{c}{K_{A}}\right)}^{n}+e^{-\beta \Delta \varepsilon_{AI}}{\left(1+\frac{c}{K_{I}}\right)}^{n}}\frac{2R}{N_{NS}}e^{-\beta \Delta \varepsilon_{R}}{)}^{-1}, \end{array}$$ (4)

where *c* is the concentration of inducer, *n* is the number of inducer binding sites on the repressor, *K*_*A*_ and *K*_*I*_ are the dissociation constants of the inducer and repressor when the repressor is in its active or inactive state, respectively, and Δ*ε*_*AI*_ is the difference in free energy between the repressor’s active and inactive states. In Ref. [48] we determined that these values are $K_{A}=139_{-22}^{+29}\;\mu M$, $K_{I}=0.53_{-0.04}^{+0.04}\;\mu M$, and Δ*ε*_*AI*_ = 4.5 *k*_*B*_*T* for LacI with the inducer IPTG. Where noted, superscripts and subscripts indicate the upper and lower bounds for the 95th percentile of the parameter value distributions. There are *n* = 2 inducer binding sites on each LacI dimer. We note that while we use Eq 4 here to represent induction of LacI by IPTG, this equation is general and can be used for any inducible system that utilizes simple repression.

**Fig 4:**
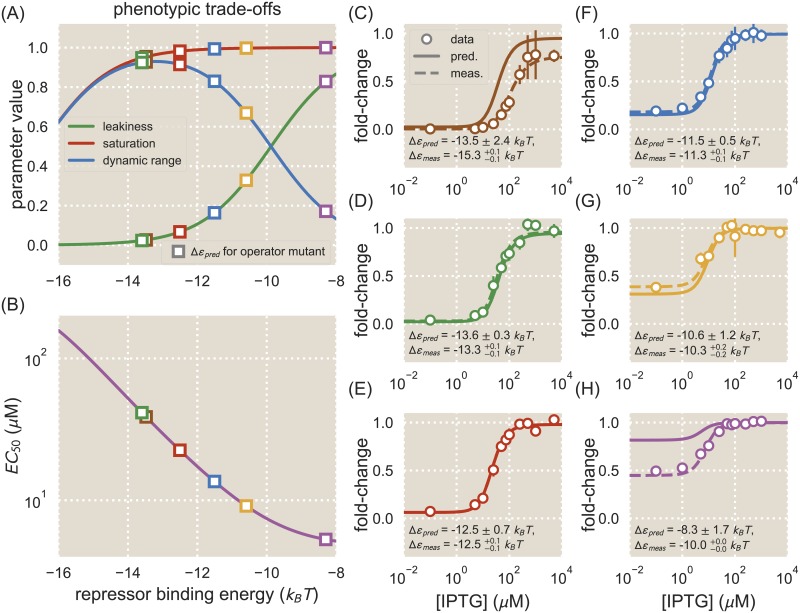
Energy matrix predictions can be used to design phenotypic responses. Phenotypic parameters exhibit trade-offs as Δ*ε*_*R*_ is varied. A: The values of the leakiness, saturation, and dynamic range are plotted as a function of transcription factor binding energy, Δ*ε*_*R*_, for a strain with repressor copy number *R* = 130. Different values of Δ*ε*_*R*_ exhibit combinations of different phenotypic properties. Several operators were chosen whose predicted binding energies (squares) result in a range of phenotypes. B: The value of the [*EC*_50_] is plotted as a function of Δ*ε*_*R*_ for a strain with *R* = 130. The [*EC*_50_] decreases as the value of Δ*ε*_*R*_ increases. C-H: Operators with different values of Δ*ε*_*R*_ were chosen to have varying induction responses based on the phenotypic trade-offs shown in (A) and (B). The fold-change is shown for each operator as IPTG concentrations are varied. The fold-change data are overlaid with the predicted induction curve (solid) and an induction curve plotted using the measured binding energy for the operator (dashed). Shown are the predicted binding energy (where the error represents the standard deviation of predictions) and the fitted binding energy (where the superscripts and subscripts represent the 95% confidence intervals of the fits).

We can use these parameter values for the *lac*-based system considered here to explore how tuning the operator-repressor binding energy Δ*ε*_*R*_ can alter the induction response when an effector (i.e. IPTG) is introduced to the system. Importantly, our sequence-energy mapping provides a straightforward avenue for tuning Δ*ε*_*R*_ by altering the binding sequence rather than mutating the repressor itself, which is much more difficult to characterize. We note that an induction response can be described by a number of key phenotypic parameters. The leakiness is the minimum fold-change when no inducer is present, given by fold- change(*c* → 0) (Eq 1 in S6 Text). The saturation is the maximum fold-change when inducer is present at saturating concentrations, given by fold- change(*c* → ∞) (Eq 2 in S6 Text). The dynamic range is the difference between the saturation and leakiness, and represents the magnitude of the induction response (Eq 4 in S6 Text). The [*EC*_50_] is the inducer concentration at which the fold-change is equal to the midpoint of the induction response (Eq 6 in S6 Text). Full expressions for these parameters are shown in S6 Text. Fig 4A and 4B show how these phenotypic parameters vary with Δ*ε*_*R*_ given the values of *K*_*A*_, *K*_*I*_, and Δ*ε*_*AI*_ listed above and the repressor copy number *R* = 130. We can see that there are inherent trade-offs between phenotypic parameter values. For instance, in this particular system one cannot tune Δ*ε*_*R*_ to obtain a small dynamic range (e.g. a dynamic range of 0.1) while also having an intermediate leakiness value (e.g. a leakiness of 0.4). Rather, one must design an induction response by choosing from the available phenotypes, or else alter the system by tuning additional parameters such as *K*_*A*_ and *K*_*I*_, which requires mutating the protein itself or using a different transcription factor altogether as in Ref. [41].

To show how energy matrices can be used to design specific induction responses, we chose six of our single base-pair mutants with a range of predicted binding energies, which we could expect to exhibit a range of phenotypic properties as shown in Fig 4A and 4B. Induction responses for each of these mutants were measured by growing cultures in the presence of varying IPTG concentrations and measuring the fold-change at each concentration. Fig 4C–4H shows how the induction data compare against theory curves plotted using the mean Δ*ε*_*R*_ values predicted from the energy matrices derived from Sort-Seq data with the O1 reference sequence and repressor copy number *R* = 130. In general the predicted induction curves match well with the data, though the predicted induction curves in Fig 4C and 4H are noticeably dissimilar to the data. Theory curves plotted using the measured binding energy (rather than the predicted binding energy) describe the data well, indicating that any mis-match between the data and the predicted theory curve is due to error in the predicted binding energy, which may arise from errors in the matrices themselves or the inferred scaling factor.

### Analysis of amino acid-nucleotide interactions

Predictive energy matrices offer a simple way of analyzing direct interactions between amino acids and nucleotides. Mutating individual amino acids in the repressor’s DNA-binding domain and then observing changes in the energy matrix makes it possible to determine how changing the amino acid composition of the DNA-binding domain alters sequence preference. If sequence specificity is altered only for specific base pairs when an amino acid is mutated, this may indicate that the amino acid interacts directly with those base pairs. While it is possible to obtain such information using binding assays [49] or labor-intensive structural biology approaches, Sort-Seq makes it possible to efficiently sample protein-DNA interactions. To analyze the effects of amino acid mutations on sequence specificity, we chose mutations which had previously been found to alter LacI-DNA binding properties without entirely disrupting the repressor’s ability to bind DNA [49, 50]. We performed Sort-Seq using strains containing one of three LacI mutants, Y20I, Q21A, or Q21M, where the first letter indicates the wild-type amino acid, the number indicates the amino acid position, and the last letter indicates the identity of the mutated amino acid. Here we used matrices derived from libraries in which only the operator was mutated; each matrix was scaled such that the average mutation carried a binding penalty equal to the average binding penalty for wild-type LacI, measured in *k*_*B*_*T* energy units.

**Fig 5:**
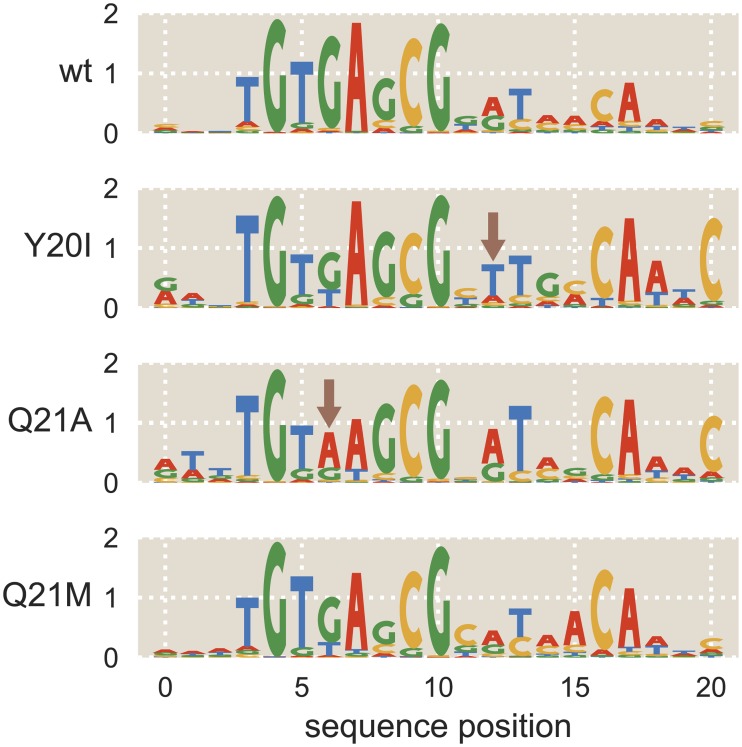
Mutations to LacI DNA-binding domain cause subtle changes to sequence specificity. Mutations were made to residues 20 and 21 of LacI, both of which lie within the DNA-binding domain. The mutations Y20I and Q21A weaken the repressor-operator binding energy, while the mutation Q21M strengthens the binding energy [50]. The sequence preferences of each mutant are represented as sequence logos. Y20I exhibits minor changes to specificity in low-information regions of the binding site, and Q21A experiences a change to specificity within a high-information region of the binding site (see arrows). Specifically, Q21A prefers A at operator position 6 while the wild-type repressor prefers G at this position.

Sequence logos derived from the mean energy matrices for each LacI mutant are shown in Fig 5, along with the wild-type sequence logo for comparison. As with the wild-type repressor, for each of the mutant repressors we find that the left half-site of the sequence logo has a stronger sequence preference. For both Y20I and Q21M, the same sequence is preferred in the left half-site as for the wild-type LacI. This contrasts with the results from Ref. [49], in which it was found that Y20I prefers an adenine at sequence position 6, rather than the guanine preferred at this position by the wild-type repressor. As in Ref. [49], we find that an adenine is preferred at sequence position 6 for the Q21A mutant. Additionally, when comparing the left and right half-sites of each energy matrix, we find that for each mutant the preferred sequence is not entirely symmetric. This is especially notable at positions 8 and 12, in which symmetry is broken in each of the sequence logos shown in Fig 5. For wild-type LacI, we see that G is preferred at position 8 with a probability of 0.75 ± 0.05, where the error represents the standard deviation of probabilities derived from each replicate matrix. At position 12, A is preferred with a probability of 0.49 ± 0.06. We see this trend repeated for Y20I, where G is 0.79 ± 0.09 probable at position 8 and T is 0.63 ± 0.10 probable at position 12, Q21A where G is 0.86 ± 0.06 probable at position 8 and A is 0.63 ± 0.05 probable at position 12, and Q21M where G is 0.61 ± 0.07 probable at position 8 and A is 0.50 ± 0.11 probable at position 12. For each mutant, these sites are found to prefer asymmetrical bases with p-value < 0.05. Thus we see that the *lac* repressor’s notable preference for a pseudo-symmetric operator is preserved in each of the mutants we tested [29, 30, 37].

### Binding site context can influence a transcription factor’s binding specificity

In this work we have used the *lac* system to demonstrate how Sort-Seq can be used to map binding site sequence to binding energy, and we used these mappings to rationally design novel genetic circuit elements and identify the effects of amino acid mutations on LacI’s sequence specificity. Importantly, this approach is not specific to the *lac* system and can be applied to any system in which transcription factors alter gene expression by binding to DNA within the promoter region. In Ref. [26] we showed how Sort-Seq could be used alongside mass spectrometry to determine the locations of transcription factor binding sites in a promoter of interest and identify which transcription factors bind to these sites. We generated energy matrices for a number of transcription factors (e.g. RelBE, MarA, PurR, XylR, and others). Here we analyze selected energy matrices from Ref. [26] to show how energy matrices can be used to understand transcriptional activity in promoters with varied architectures beyond simple repression.

One of the questions we wish to answer is to what extent altering the context of a binding site within a regulatory architecture will alter sequence specificity. One hypothesis is that a transcription factor’s preferred binding sequence will remain the same regardless of how its binding site is positioned within the regulatory architecture. However, it is known that factors beyond the core operator sequence, such as flanking sequences and DNA shape, can affect sequence specificity [19, 51, 52]. Additionally, interactions with other proteins may alter the way a transcription factor contacts the DNA, which could affect sequence specificity as well [53]. It is important to know whether a transcription factor’s specificity is sensitive to the context of the binding site within the promoter architecture, as this determines the extent to which an energy matrix can be used to analyze binding sites throughout the genome. Additionally, observing how sequence specificities change with binding site context may alert us to changes in regulatory mechanisms as the operator is moved to different positions in the promoter.

**Fig 6:**
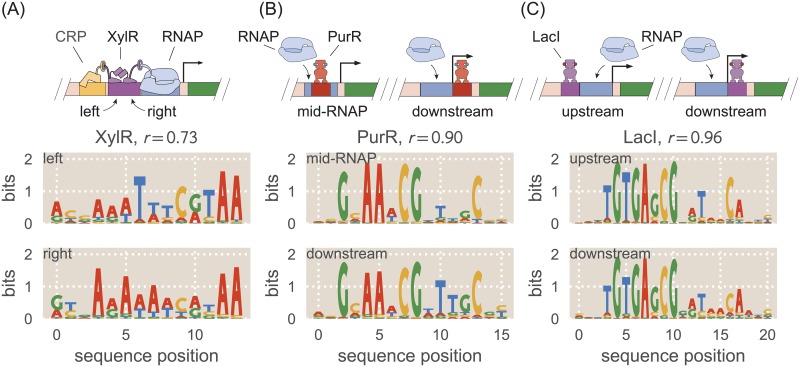
Regulatory context can alter sequence preference. Sequence logos were obtained for the same transcription factors in different regulatory contexts and compared against one another. The Pearson’s correlation coefficient *r* between energy matrices is noted for each pair of binding sites. A: Sequence logos are shown for the two adjacent binding sites for the activator XylR in the *xylE* promoter, shown schematically at top. The sequence logos for the two binding sites indicate that they have significantly different sequence preferences. B: Sequence logos are shown for the PurR binding site in the *purT* promoter and a PurR binding site for a synthetic simple repression promoter in which the binding site is positioned differently, shown schematically at top. The sequence logos for the two binding sites indicate nearly identical sequence preferences. C: Sequence logos are shown for a LacI binding site upstream of the RNAP binding site and a LacI binding site downstream of the RNAP. Although regulatory mechanisms differ between these two binding sites, their sequence logos are nearly identical.

In Ref. [26], we used Sort-Seq to obtain energy matrices and sequence logos for the transcription factors XylR and PurR in the context of the natural promoters for *xylE* and *purT*, respectively. The *xylE* promoter has two XylR binding sites directly adjacent to one another, allowing us to compare these two energy matrices against each other. In this context, we find that XylR appears to act as an activator in tandem with a CRP binding site. Sequence logos for the two XylR binding sites are shown in Fig 6A, obtained from mean energy matrix values for each site. The energy matrices and sequence logos for these binding sites have some significant dissimilarities. Dissimilarities are particularly notable at positions 6-8, where the left-hand site prefers “TTT” with probabilities 0.89 ± 0.03, 0.54 ± 0.01, and 0.65 ± 0.03 respectively, and the right-hand site prefers “AAA” with probabilities 0.58 ± 0.01, 0.79 ± 0.01, and 0.47 ± 0.01. We can say that the sequence preferences at each of these positions differ between the two binding sites with p-value < 0.0001. These changes in sequence specificity may be due to interactions with neighboring DNA-binding proteins. We note that in the *xylE* promoter the left-hand XylR site is adjacent to a CRP site, while the right-hand XylR site is adjacent to the RNAP site. The close proximity of these binding sites suggests that there may be direct interactions between proteins. Such interactions could constrain the binding conformations of each XylR copy and thus alter how each XylR interacts with its DNA binding site. The Pearson’s correlation coefficient *r* between the mean values of the two energy matrices is *r* = 0.73.

In Ref. [26] we find that PurR acts as a repressor in the *purT* promoter, with a single binding site between the -10 and -35 sites. In order to compare the associated energy matrix with a PurR energy matrix from a different regulatory context, here we create a synthetic promoter in which the PurR binding site has been moved directly downstream of the RNAP site. This should continue to be a simple repression architecture in which repressor binding occludes RNAP binding, but the change in operator position may alter the repressor’s interaction with the DNA. Sequence logos for both PurR binding sites are shown in Fig 6B. The two PurR sequence logos are very similar to one another, indicating no significant changes in the interactions between the repressor and the DNA. We calculate the Pearson’s correlation coefficient between the two energy matrices to be *r* = 0.90, which is significantly higher than the value calculated for the two XylR energy matrices.

We additionally performed Sort-Seq on a LacI simple repression construct in which the *lac* operator was placed upstream of the RNAP binding site rather than downstream. In Ref. [27] it is shown that LacI binding to an upstream operator still represses, but whereas a downstream operator represses by preventing RNAP from binding, an upstream operator appears to directly contact a bound RNAP and prevent it from escaping the promoter. Moreover, an upstream operator’s binding strength does not directly correspond with the level of repression associated with the promoter. These factors make repression by an upstream *lac* operator an interesting architecture to compare with repression by a downstream *lac* operator. Sequence logos for the upstream and downstream LacI binding sites are shown in Fig 6C. These logos are very similar to one another, despite the fact that the repression mechanisms and protein interactions differ for these two architectures. The Pearson’s correlation coefficient between the two matrices is *r* = 0.96.

For the above analysis we note that the energy matrices used to make these sequence logos were scaled using a theoretical “average” binding penalty derived from a statistical mechanical analysis of transcriptional regulation (see S2 Text). This is because a number of the necessary parameters for some of these architectures, such as repressor copy number or wild-type binding energies, still remain poorly characterized. In order to confidently infer scaling factors for transcription factor binding sites, it is necessary to have a thermodynamic model that is known to accurately describe the architecture and contains a minimum of unknown parameter values. For the example of simple *lac* repression, our thermodynamic model has been shown repeatedly to accurately describe the key mechanisms of simple repression architectures [24, 32, 48, 54, 55]. We have performed our Sort-Seq experiments in strains which are known to lack any additional LacI binding sites in the chromosome, in which we know the repressor copy number *R*, and which we know do not contain any known inducers of LacI. These factors allow us to confidently infer energy matrix scaling factors.

If we consider the case of simple repression by PurR, we know that the promoter is under co-repression through concerted binding of the ligand hypoxanthine to PurR [56]. While Eq (4) provides a rational model for a simple allosteric architecture of this type, the allosteric parameters *K*_*A*_, *K*_*I*_, and *ε*_*AI*_ are uncharacterized; moreover, the concentration of inducer in the system is uncharacterized. While the PurR repressor copy number has been measured to be about 400 dimers per cell under the growth conditions considered here [39], attempting to fit the remaining parameters with our Sort-Seq expression data is not possible due to significant degeneracy in the parameter space [48].

Note that in the case of the XylR architecture, we are hesitant to suggest a new thermodynamic model for *p*_*bound*_ without further work to understand the specific mechanism of regulation and identify all of the relevant parameters. For example, it is unclear whether a single bound copy of XylR is capable of activating the promoter, or whether it must bind in a complex with another copy of XylR. Additionally, there are likely to be physical interactions between CRP, the two copies of XylR, and RNAP that would be represented by effective interaction energy parameters. This would add a significant number of parameters to the thermodynamic model, all with unknown values, which would make it difficult to make accurate estimates of any parameter value in the model. This prevents us from inferring scaling factors for the XylR binding sites using MCMC.

## Discussion

In this work, we apply quantitative modeling to *in vivo* experimental data to analyze interactions between transcription factors and their binding sites under multiple conditions. As an example of how our approach might be used to analyze a transcription factor’s sequence-specific binding energy, we used Sort-Seq to create energy matrices that map DNA sequence to binding energy for the *lac* repressor (Fig 2). We performed this work in the context of a simple repression architecture, which is widespread among bacterial promoters [57] and is frequently used in synthetic biology [47, 58, 59]. We test our model’s predictions against binding energies inferred from fold-change measurements of roughly 30 *lac* operator mutants (Fig 3). These predictions proved to be accurate to within ∼ 1.0 *k*_*B*_*T* for binding sites with up to four mutations relative to the reference sequence. We note, however, that our energy matrices cannot be used to predict the binding energies of operator mutants containing insertions or deletions, such as the synthetic operator Oid.

Because we are able to accurately predict operator binding energies, our sequence-energy mappings can be used to design specific regulatory responses, which is of great utility to synthetic biology. We combine energy matrices with a thermodynamic model of inducible simple repression to design induction curves, as demonstrated in Fig 4 [48]. We note that in spite of the overall success of our predictions, there remain some predictions that are significantly different from the measured values (see the outliers in Fig 3C). Such inaccuracies are particularly problematic when using energy matrices for design applications, as discrepancies between a system’s expected and actual response may render a designed system unsuitable for its intended application. We can see examples of this in Fig 4F–4H, where inaccuracies in binding energy predictions are reflected in the predicted titration curves. Two of the six prediction curves do not accurately describe the data, with the data exhibiting mis-matches in either saturation or leakiness. If the saturation or leakiness are vital parameters in the designed system, then such a mis-match could cause the system to fail. Importantly, the error associated with binding energy predictions varies depending on the method used to obtain a scaling factor to convert the energy matrix to absolute energy units. As discussed in S2 Text, inferring the scaling factor using MCMC, as we do for our predictions in the main text, introduces a source of error related to uncertainties in the inference procedure. Error can be reduced by using an alternative scaling procedure such as fitting to sequences with known binding energies.

We also explore how sequence specificity is altered when transcription factor amino acids are mutated. To do this, we repeat our Sort-Seq experiments in bacterial strains expressing LacI mutants in which the DNA-binding domain has been altered (Fig 5). Because all nucleotides in the binding site are mutated with some frequency in Sort-Seq experiments, we are able to identify changes in specificity throughout the entire binding site. Other methods for analyzing the sequence preference of transcription factor mutants tend to be more laborious and less fine-grained, often focusing on a small set of nucleotides within the binding site. These include binding experiments between DNA mutants and protein mutants [49], gene expression experiments using chimeric transcription factor proteins [60], and comparative genomics [61].

We further explore how regulatory context alters sequence specificity. We generate sequence logos from energy matrices obtained for the transcription factors XylR, PurR, and LacI in different regulatory contexts, as shown in Fig 6. We find that the two adjacent XylR binding sites exhibit significantly different binding specificities, possibly due to interactions between transcription factors. In contrast, the simple repression constructs analyzed for PurR and LacI have nearly identical sequence specificities. By itself, our method is unable to determine the causes of context-dependent changes in sequence specificity, though it is known that DNA shape or binding to cofactors can alter a transcription factor’s specificity [51–53]. Rather, our approach can be used to determine whether a given binding site’s sequence preferences diverge from the “standard” sequence specificity for the relevant transcription factor, and further experiments (such as SELEX-seq in the presence of a transcription factor and possible cofactors [53]) can be performed to determine the cause of the change in sequence specificity.

A major advantage of our *in vivo* approach is that it allows us to analyze transcription factors in their natural context, in the presence of interacting proteins, small molecules, and DNA shape effects. This is especially important when analyzing regulatory regions that have not been previously annotated, as was the case for the XylR and PurR matrices obtained in Ref. [26]. However, a clear advantage of *in vitro* approaches is that they can accurately measure low-affinity binding sites [12, 13, 15]. When using our *in vivo* approach, weaker reference sequences produce energy matrices with variable quality and are more likely to make poor predictions (see S4 Text). However, accuracy may be improved by investigating ways to reduce the experimental noise associated with *in vivo* systems, for instance by incorporating promoter constructs as single copies in the chromosome rather than multiple copies on plasmid, for example using the “landing pad” technique described in Ref. [62]. We further note that the primary methodology used here, in which an energy matrix scaling factor is inferred via MCMC, offers an indirect readout of transcription factor binding energies that relies on the assumption that transcription will always be initiated (at some rate) if the promoter is occupied by RNAP. While this assumption often provides a valid approximation of the mechanisms of transcription, previous work has shown that this is not always an appropriate model [24, 25]. More detailed models of transcription would be required in order to apply this methodology to systems in which RNAP occupancy does not reliably predict transcription initiation. When such models are not available, energy matrix scaling factors can be inferred using a theoretical binding energy penalty for mutations as detailed in S2 Text.

This work provides a foundation for further studies that would benefit from sequence-energy mappings. For example, our analysis of three LacI amino acid mutants could be expanded to include a full array of LacI DNA-binding mutants, which would allow one to make inferences regarding repressor-operator coevolution. Additionally, while we make extensive use of LacI in the present work, similar analyses could be performed with any transcription factor, making it possible to improve upon the genomically-inferred sequence logos presently available for many transcription factors. We demonstrate this capability in Fig 6, where we identify the sequence specificities of two additional transcription factors, XylR and PurR. The data shown in Fig 6 further demonstrate that we can assess effects on sequence specificity that result from factors such as binding site positioning or protein-protein interactions. Thus, for cases in which it is known that sequence specificity is affected by DNA shape, flanking sequences, cofactor binding, or other factors outside of the operator binding sequence, our approach can be used to obtain a base-pair resolution map of the effects on sequence specificity.

Finally, we note that one of the primary strengths of our approach is that it can be used to elucidate the transcriptional regulation of a gene with a previously-unknown regulatory architecture. As shown in Ref. [26], Sort-Seq can be combined with mass spectrometry to identify transcription factor binding sites and those sites’ regulatory roles for any gene of interest. Here we show that data sets obtained in this manner can also be used to map sequence to binding energy, thus showing that a single experiment can be used to characterize multiple aspects of a previously-unannotated regulatory sequence. Currently, we convert our energy matrices to absolute energy units using a theoretical scaling factor for cases where thermodynamic models for the regulatory architecture are unknown, but future work may improve our ability to identify thermodynamic models and thus infer more accurate scaling factors. Furthermore, our approach does not rely specifically on the Sort-Seq technique used here, but can be adapted to multiple experimental designs, such as RNA-seq based MPRAs that have been demonstrated in multiple model systems [7, 63–65]. Over time, we envision incorporating high-throughput synthesis and analysis techniques to adapt our approach for genome-wide studies in both prokaryotes and eukaryotes.

## Methods

### Sort-Seq libraries

To generate promoter libraries for Sort-Seq, mutagenized oligonucleotide pools were purchased from Integrated DNA Technologies (Coralville, IA). These consisted of single-stranded DNA containing the *lacUV5* promoter and LacI operator plus 20 bp on each end for PCR amplification and Gibson Assembly. Either both the *lacUV5* promoter and LacI binding site or only the LacI binding site was mutated with a ten percent mutation rate per nucleotide. These oligonucleotides were amplified by PCR and inserted back into a pUA66-operator-GFP construct using Gibson Assembly. To achieve high transformation efficiency, reaction buffer components from the Gibson Assembly reaction were removed by drop dialysis for 90 minutes and cells were transformed by electroporation of freshly prepared electrocompetent cells. Following an initial outgrowth in SOC media, cells were diluted with 50 mL LB media and grown overnight under kanamycin selection. Transformation typically yielded 10^6^ − 10^7^ transformants as assessed by plating 100 *μ*L of cells diluted 1:10^4^ onto an LB plate containing kanamycin and counting the resulting colonies.

### DNA constructs for fold-change measurements of mutant operators

Simple repression motifs used in fold-change measurements were adapted from those in Garcia *et al*. [32]. Briefly, a simple repression construct with the O1 operator sequence was cloned into a pZS25 plasmid background directly downstream of a *lacUV5* promoter, driving expression of a YFP gene when the operator is not bound by LacI. This plasmid contains a kanamycin resistance gene for selection. Mutant LacI operator constructs (listed in Table 1) were generated by PCR amplification of the *lacUV5* O1-YFP plasmid using primers containing the point mutations as well as sufficient overlap for re-circularizing the amplified DNA by one-piece Gibson Assembly.

**Table 1:** Mutant operator sequences. The listed operator sequences were used to evaluate energy matrix predictions. They are mutated relative to the O1 *lac* operator. The predicted binding energy was generated using the matrix with an O1 reference sequence with *R* = 130 LacI tetramers in the background strain.

Sequence	Predicted Δ*ε*_*R*_ (*k*_*B*_*T*)	Measured Δ*ε*_*R*_ (*k*_*B*_*T*)
**1 bp mutants**:		
AATTGTGAGCGGAGAACAATT	-12.63	-12.24
AATTGTGAGCGCATAACAATT	-15.71	-15.30
AATTGTGAGCGGATCACAATT	-15.22	-14.99
AATTGTGAGCGGAAAACAATT	-12.91	-12.50
AATTGCGAGCGGATAACAATT	-12.14	-11.30
AATTGTGAGGGGATAACAATT	-13.16	-12.35
AATTGTGAGCGGATATCAATT	-13.66	-13.29
AATTGTGAGCAGATAACAATT	-11.11	-10.25
AATTGTGAGAGGATAACAATT	-8.89	-10.00
**2 bp mutants**:		
AATTGTGAGCGGGTAACAACT	-13.82	-14.79
AAATGTGAGCGGATAACAACT	-13.61	-14.40
AATTGTGAGCGAGTAACAATT	-14.36	-15.12
ATTTGTGAGCGGAGAACAATT	-12.55	-11.52
CATTGTGAGCGCATAACAATT	-15.34	-14.80
AATTGTGAGCGGAACACAATT	-12.83	-13.31
AATTGTGAGCGGAATACAATT	-11.70	-12.03
AATTGCGAGCGGATAACAAAT	-12.06	-10.78
AATTGTGAGGGGATAACAATC	-14.13	-12.15
**3 bp mutants**:		
AAATGTGAGCGAGTAACAATT	-13.84	-14.57
AATTGTGAGCGAGTAACAACT	-13.19	-14.67
ATTTGTGAGCGAAGAACAATT	-11.92	-10.83
CATTGTGAGCGCATAACATTT	-15.39	-14.18
AATTGTGAGCGGAACACAATG	-13.72	-12.17
AATTGTGAGCGGGATACAATT	-11.39	-11.86
AATTGCGAGCGGATAACAAAG	-12.96	-10.62
AATTGTGAGGGTATAACAATC	-14.10	-11.79

A second construct was generated to express LacI at a specified copy number. Specifically, *lacI* was cloned into a pZS3*1 background that provides constitutive expression of LacI from a P_LtetO−1_ promoter [66]. This plasmid contains a chloramphenicol resistance gene for selection. The LacI copy number is controlled by mutating the ribosomal binding site (RBS) for the *lacI* gene as described in [67] using site-directed mutagenesis (Quickchange II; Stratagene, San Diego, CA) and further detailed in [32]. Here, we mutated the RBS such that it would produce a LacI copy number of ∼130 tetramers once the construct had been integrated into the chromosome.

Once the plasmids had been generated, the promoter and *lacI* constructs were each amplified by PCR and integrated into the chromosome by lambda-red recombineering using the pSIM6 expression plasmid [68]. The promoter construct and YFP gene were inserted into the *galK* locus in the *E. coli* genome and the *lacI* construct was inserted into the *ybcN* locus.

### Construction of LacI amino acid mutants

As previously mentioned, wild-type *lacI* was cloned into a pZS3*1 background providing constitutive expression of LacI, with the LacI copy number mediated by a mutated RBS. We used the RBS corresponding to a LacI tetramer copy number of ∼130 for each mutant. To create DNA-binding mutants for LacI we used site-directed mutagenesis (Quickchange II; Stratagene, San Diego, CA) using the mutagenesis primers listed in Table 2. We mutated the amino acid Y to I at position 20 and Q to A or M at position 21. We chose these mutations based on data from previous studies [49, 50], though we note that our amino acid numbering system is shifted by +3 relative to the mutants in these previous studies since we use a slightly different version of *lacI*. As with the wild-type *lacI*, we integrate the mutants into the genome at the *ybcN* locus by lambda-red recombineering using the pSIM6 expression plasmid.

**Table 2:** Primers used in this work. The listed primer sequences were used to generate plasmids for Sort-Seq experiments or for use in creating strains with mutated operators or LacI.

Name	Sequence	Comments
lac_ins_fwd	CCCTTTCGTCTTCAC	Used to amplify *lac* promoter insert for Gibson
lac_ins_rev	CCTTTACTCATATGTATATCTCCTTTTAAATCTAGAGGAT	Used to amplify *lac* promoter insert for Gibson
pUA66_frameshift_fwd	GATATACATATGAGTAAAGGAGAAGAACTT	Used to amplify pUA66 vector for Gibson
pUA66_rev	TCGAGGTGAAGACGAAAG	Used to amplify pUA66 vector for Gibson
GCMWC-001_Q21_rev	CCGGCATACTCTGCGACA	Mutagenesis primer for LacI residue 21
GCMWC-002_Q21M	GTGTCTCTTATATGACCGTTTCCCGC	Mutagenesis primer for LacI residue 21 Q → M
GCMWC-003_Q21A	TGTCTCTTATGCGACCGTTTCCCGC	Mutagenesis primer for LacI residue 21 Q → A
GCMWC-009_Y20_rev	GCATACTCTGCGACATCGTATAAC	Mutagenesis primer for LacI residue 20
GCMWC-010_Y20I	CGGTGTCTCTATTCAGACCGTTTC	Mutagenesis primer for LacI residue 20 Y → I

### Bacterial strains

*E. coli* strains used in this work were derived from K12 MG1655. To generate strains with different LacI copy number, the *lacI* constructs were integrated into a strain that additionally has the entire *lacI* and *lacZYA* operons removed from the chromosome. These constructs were integrated at the *ybcN* chromosomal location. This resulted in strains containing mean LacI tetramer copy numbers of *R* = 11 ± 2, 30 ± 10, 62 ± 15, 130 ± 20, 610 ± 80, and 870 ± 170, where the error denotes the standard deviation of at least three replicates as measured by quantitative western blots in Ref. [32].

For Sort-Seq experiments, plasmid promoter libraries were constructed as described above and then transformed into the strains with *R* = 30, 62, 130 or 610. For fold-change measurements, each O1 operator mutant was integrated into strains containing each of the listed LacI copy numbers. These simple repression constructs were chromosomally integrated at the *galK* chromosomal location via lambda red recombineering. Generation of the final strains containing a simple repression motif and a specific LacI copy number was achieved by P1 transduction. For each LacI titration experiment, we also generated a strain in which the operator-YFP construct had been integrated, but the *lacI* and *lacZYA* operons had been removed entirely. This provided us with a fluorescence expression measurement corresponding to *R* = 0, which is necessary for calculation of fold-change.

### Sort-Seq fluorescence sorting

For each Sort-Seq experiment, cells were grown to saturation in lysogeny broth (LB) and then diluted 1:10,000 into minimal M9 + 0.5% glucose for overnight growth. Once these cultures reached an OD of 0.2-0.3 the cells were washed three times with PBS by centrifugation at 4000 rpm for 10 minutes at 4°C. They were then diluted two-fold with PBS to reach an approximate OD of 0.1-0.15. These cells were then passed through a 40 *μ*m cell strainer to eliminate any large clumps of cells.

A Beckman Coulter MoFlo XDP cell sorter was used to obtain initial fluorescence histograms of 500,000 events per library in the FL1 fluorescence channel with a PMT voltage of 800 V and a gain of 10. The histograms were used to set four binning gates that each covered ∼ 15% of the histogram. 500,000 cells were collected into each of the four bins. Finally, sorted cells were regrown overnight in 10 mL of LB media, under kanamycin selection.

### Sort-Seq sequencing and data analysis

Overnight cultures from each sorted bin were miniprepped (Qiagen, Germany), and PCR was used to amplify the mutated region from each plasmid for Illumina sequencing. The primers contained Illumina adapter sequences as well as barcode sequences that were unique to each fluorescence bin, enabling pooling of the sorted samples. Sequencing was performed by either the Millard and Muriel Jacobs Genetics and Genomics Laboratory at Caltech or NGX Bio (San Fransisco, CA). Single-end 100bp or paired-end 150bp flow cells were used, with about 500,000 non-unique sequences collected per library bin. After performing a quality check and filtering for sequences whose PHRED score was greater than 20 for each base pair, the total number of useful reads per bin was approximately 300,000 to 500,000 per million reads requested. Energy weight matrices for binding by LacI and RNAP were inferred using Bayesian parameter estimation with a error-model-averaged likelihood as previously described [28, 69] and further detailed in S1 Text. Unless otherwise specified, replicates were formed by randomly splitting the Sort-Seq data sets into three groups containing equal numbers of sequences. Each of these groups was used to create a separate energy matrix. These matrices were either used to create a “mean” energy matrix, as when a matrix heat-map or sequence logo is displayed, or were used individually to create replicate binding energy predictions.

Where p-values are noted for sequence preference, the probabilities of each base at a given position were first calculated for each replicate matrix using the equation

$$\begin{array}{c} {\text{prob}}_{N}=\frac{e^{-\varepsilon_{N}}}{e^{-\varepsilon_{A}}+e^{-\varepsilon_{C}}+e^{-\varepsilon_{G}}+e^{-\varepsilon_{T}}}, \end{array}$$ (5)

where *N* represents a given base, and *ε*_*N*_ is the binding energy associated with that base at the given sequence position. With these probabilities in hand, we used a one-way Welch’s ANOVA test implemented in R to determine if there was a statistically significant difference in mean probability values. If a statistical difference (p-value < 0.01) was found between the means, we confirmed that the most probable base was statistically distinct from all other bases using a Games-Howell post-hoc analysis implemented in R.

### Fold-change measurements by flow cytometry

Fold-change measurements were collected as previously described in Ref. [48] on a MACSquant Analyzer 10 Flow Cytometer (Miltenyi Biotec, Germany). Briefly, YFP fluorescence measurements were collected using 488nm laser excitation, with a 525/50 nm emission filter. Settings in the instrument panel for the laser were as follows: trigger on FSC (linear, 423V), SSC (linear, 537 V), and B1 laser (hlog, 790V). Before each experiment the MACSquant was calibrated using MACSQuant Calibration Beads (Miltenyi Biotec, CAT NO. 130-093-607). Cells were grown to OD 0.2-0.3 and then diluted tenfold into ice-cold minimal M9 + 0.5% glucose. Cells were then automatically sampled from a 96-well plate kept at approximately 4°–10°C using a MACS Chill 96 Rack (Miltenyi Biotec, CAT NO. 130-094-459) at a flow rate of 2,000–6,000 measurements per second.

For those measurements that were taken for IPTG induction curves, cells were grown as above with the addition of an appropriate concentration of IPTG (Isopropyl *β*-D-1 thiogalactopyranoside Dioxane Free, Research Products International). For each IPTG concentration, a stock of 100-fold concentrated IPTG in MilliQ-purified water was prepared and partitioned into 100 *μ*L aliquots. The same parent stock was used for all induction experiments described in this work.

The fold-change in gene expression was calculated by taking the ratio of the mean YFP expression of the population of cells in the presence of LacI to that in the absence of LacI. Since the measured fluorescence intensity of each cell also includes autofluorescence which is present even in the absence of YFP, we account for this background by computing the fold change as

$$\begin{array}{c} \text{fold-change}=\frac{\left\langle I_{R>0}\right\rangle -\left\langle I_{\text{auto}}\right\rangle }{\left\langle I_{R=0}\right\rangle -\left\langle I_{\text{auto}}\right\rangle }, \end{array}$$ (6)

where 〈*I*_*R*>0_〉 is the average cell YFP intensity in the presence of repressor, 〈*I*_*R*=0_〉 is the average cell YFP intensity in the absence of repressor, and 〈*I*_auto_〉 is the average cell autofluorescence intensity as determined by measuring the fluorescence of cells in which *R* = 0 and there is no fluorescent reporter.

## Supporting information

S1 Fig List of wild-type reporter constructs. A: Wild-type versions of reporter constructs that were used either for Sort-Seq (all) or for measuring operator mutant binding energies (simple *lac* repression). B: Wild-type versions of sequences that were inferred for PurR and XylR in Ref. [26].
(PDF)

S2 Fig Energy logos for O1, O2, and O3. Energy logos are shown for O1, O2, and O3 energy matrices displayed in Fig 2. These logos represent the mean affinity in *k*_*B*_*T* of each base at each position relative to the mean binding energy at each sequence position.
(PDF)

S3 Fig Comparison of O1 energy values from the present work and Zuo *et. al* (2015). Energy values were inferred for all bases in each position of the O1 *lac* promoter using *in vivo* methods in the present work (with reference sequence O1 and repressor copy number *R* = 130) and *in vitro* methods in Zuo *et. al* (2015). The corresponding values from each matrix are plotted against one another. We show comparisons in which our matrix is scaled using two different methods to determine the scaling factor: (A) MCMC inference and (B) least-squares fitting. Because there is some error associated with the MCMC inference procedure, we see that the error bars are larger and the comparison is somewhat worse than for the matrix scaled using least-squares fitting. Note that the values from the Zuo matrix were transformed to match the convention of our matrix by setting the wild-type sequence to 0 *k*_*B*_*T* and giving any increases in binding strength negative energy values, and decreases in binding strength positive energy values relative to the wild-type sequence.
(PDF)

S4 Fig Fold-change measurements for 1 bp mutants. Fold-change measurements are shown for nine 1 bp operator mutants in strains with *R* = 11, 30, 62, 130, 610, or 870. These measurements are overlaid with the measured (fitted) binding energy measurements for each mutant and the predicted measurements as listed in the main text. Predicted energy values are listed along with the standard deviation in predictions, and measured energy values are listed along with the 95% confidence intervals for the fitted energies. Note that the bottom three plots do not display data points for *R* = 62, as the data for these strains were outliers.
(PDF)

S5 Fig Fold-change measurements for 2 bp mutants. Fold-change measurements are shown for nine 2 bp operator mutants in strains with *R* = 11, 30, 62, 130, 610, or 870. These measurements are overlaid with the measured (fitted) binding energy measurements for each mutant and the predicted measurements as listed in the main text. Predicted energy values are listed along with the standard deviation in predictions, and measured energy values are listed along with the 95% confidence intervals for the fitted energies.
(PDF)

S6 Fig Fold-change measurements for 3 bp mutants. Fold-change measurements are shown for seven 3 bp operator mutants in strains with *R* = 11, 30, 62, 130, 610, or 870. These measurements are overlaid with the measured (fitted) binding energy measurements for each mutant and the predicted measurements as listed in the main text. Predicted energy values are listed along with the standard deviation in predictions, and measured energy values are listed along with the 95% confidence intervals for the fitted energies.
(PDF)

S1 Text Bayesian inference of energy matrix models. (PDF)

S2 Text Alternate methods for obtaining energy matrix scaling factor. (PDF)

S3 Text Comparing single-point energy matrix models with higher-order models. (PDF)

S4 Text Influence of regulatory parameters on energy matrix quality. (PDF)

S5 Text Comparison of full-promoter and operator-only energy matrix predictions. (PDF)

S6 Text Expressions for phenotypic parameters of induction responses. (PDF)

S1 Models Text files containing all energy matrix models used in this work. (GZ)
