# Supplementary material for: Mapping DNA sequence to transcription factor binding energy in vivo
Source: PLoS Comput Biol. 2019 Feb 4;15(2):e1006226. doi: 10.1371/journal.pcbi.1006226 (PMC6375646; doi:10.1371/journal.pcbi.1006226)
Supplement: S1 Fig — A: Wild-type versions of reporter constructs that were used either for Sort-Seq (all) or for measuring operator mutant binding energies (simple lac repression). B: Wild-type versions of sequences that were inferred for PurR and XylR in Ref. [26]. (PDF) [file pcbi.1006226.s001.pdf]

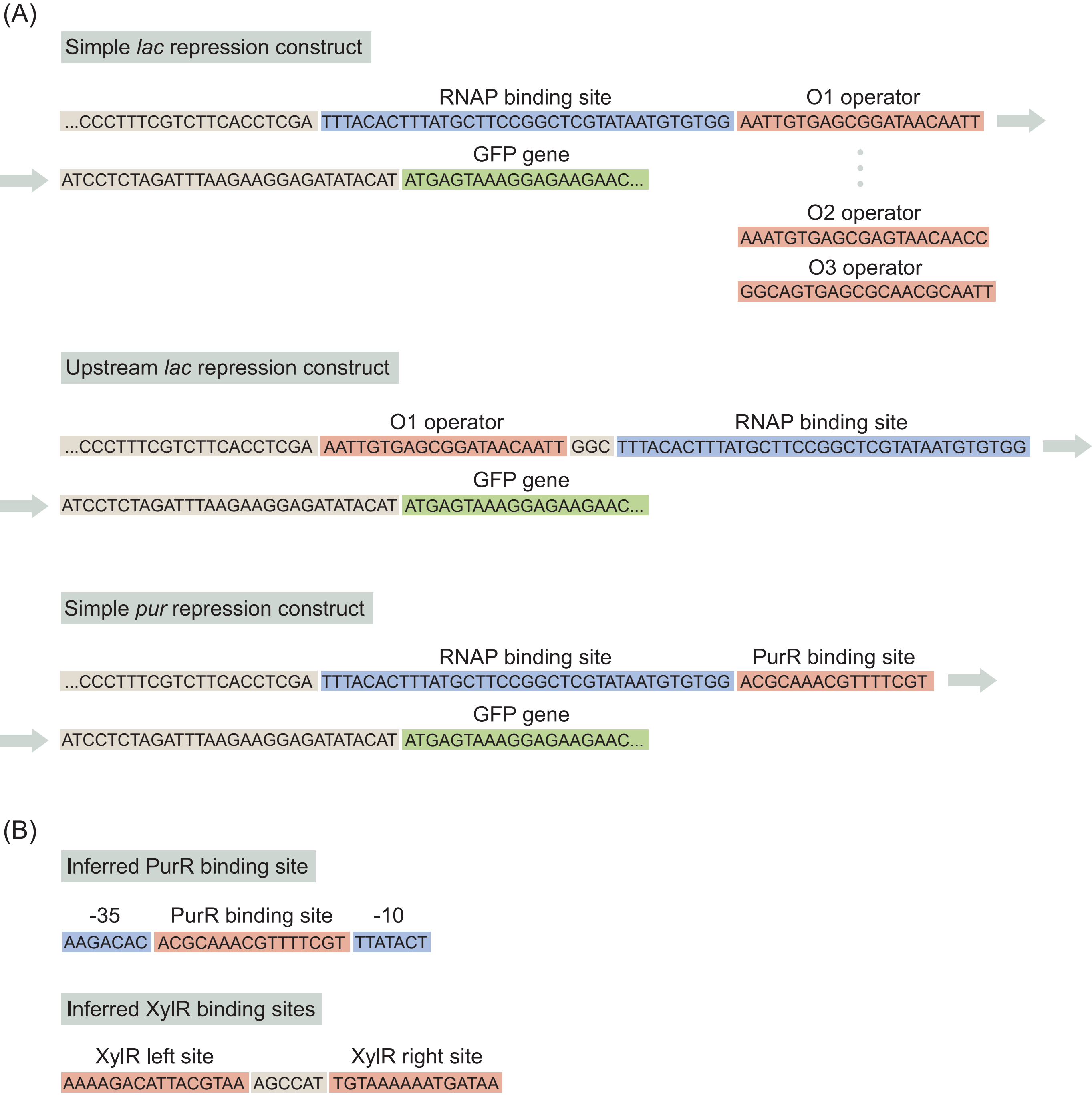
(A)
Simple lac repression construct
RNAP binding site
O1 operator
...CCCTTTCGTCTTCACCTCGA
TTTACACTTTATGCTTCCGGCTCGTATAATGTGTGG
AATTGTGAGCGGATAACAATT
GFP gene
ATCCTCTAGATTTAAGAAGGAGATATACAT
ATGAGTAAAGGAGAAGAAC...
O2 operator
AAATGTGAGCGAGTAACAACC
O3 operator
GGCAGTGAGCGCAACGCAATT
Upstream lac repression construct
O1 operator
RNAP binding site
...CCCTTTCGTCTTCACCTCGA
AATTGTGAGCGGATAACAATT
GGC
TTTACACTTTATGCTTCCGGCTCGTATAATGTGTGG
GFP gene
ATCCTCTAGATTTAAGAAGGAGATATACAT
ATGAGTAAAGGAGAAGAAC...
Simple pur repression construct
RNAP binding site
PurR binding site
...CCCTTTCGTCTTCACCTCGA
TTTACACTTTATGCTTCCGGCTCGTATAATGTGTGG
ACGCAAACGTTTTCGT
GFP gene
ATCCTCTAGATTTAAGAAGGAGATATACAT
ATGAGTAAAGGAGAAGAAC...
(B)
Inferred PurR binding site
-35
PurR binding site
-10
AAGACAC
ACGCAAACGTTTTCGT
TTATACT
Inferred XylR binding sites
XylR left site
XylR right site
AAAAGACATTACGTAA
AGCCAT
TGTAAAAAATGATAA
