# Supplementary figures and images for: Mapping DNA sequence to transcription factor binding energy in vivo
Source: PLoS Comput Biol. 2019 Feb 4;15(2):e1006226. doi: 10.1371/journal.pcbi.1006226 (PMC6375646; doi:10.1371/journal.pcbi.1006226)

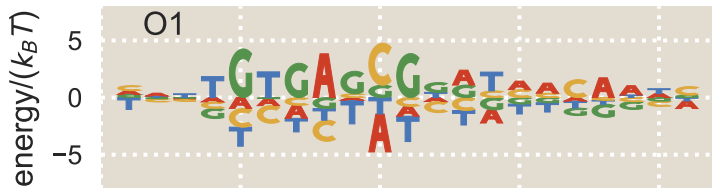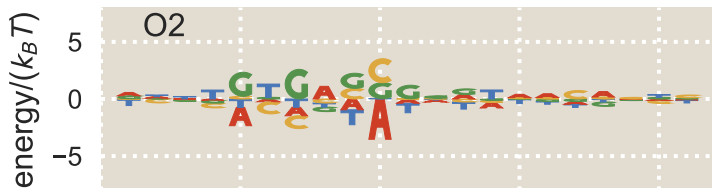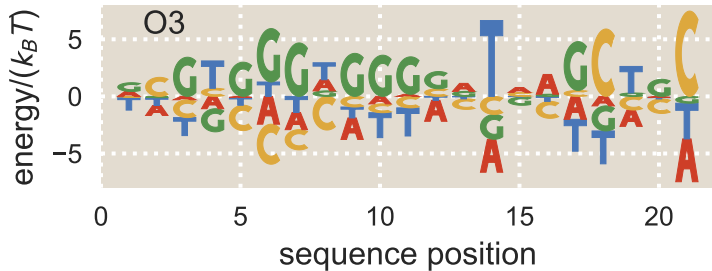

Supplement: S2 Fig — Energy logos are shown for O1, O2, and O3 energy matrices displayed in Fig 2. These logos represent the mean affinity in kBT of each base at each position relative to the mean binding energy at each sequence position. (PDF) [file pcbi.1006226.s002.pdf]

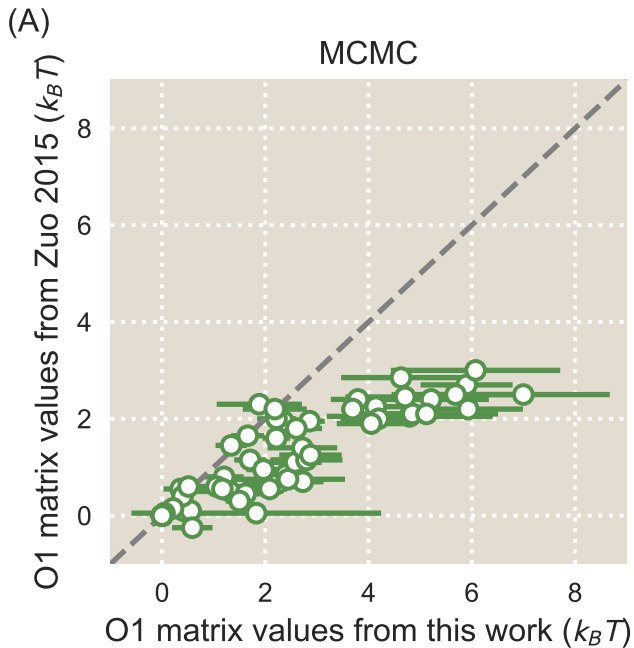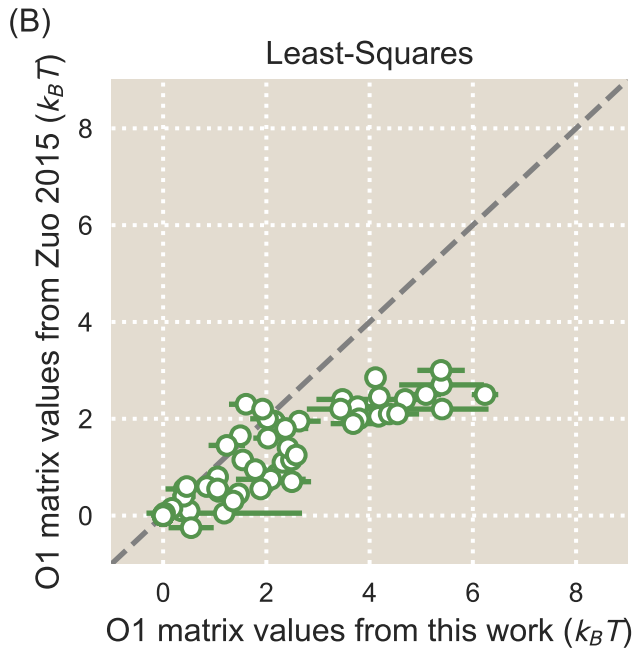

Supplement: S3 Fig — Energy values were inferred for all bases in each position of the O1 lac promoter using in vivo methods in the present work (with reference sequence O1 and repressor copy number R = 130) and in vitro methods in Zuo et. al (2015). The corresponding values from each matrix are plotted against one another. We show comparisons in which our matrix is scaled using two different methods to determine the scaling factor: (A) MCMC inference and (B) least-squares fitting. Because there is some error associated with the MCMC inference procedure, we see that the error bars are larger and the comparison is somewhat worse than for the matrix scaled using least-squares fitting. Note that the values from the Zuo matrix were transformed to match the convention of our matrix by setting the wild-type sequence to 0 kBT and giving any increases in binding strength negative energy values, and decreases in binding strength positive energy values relative to the wild-type sequence. (PDF) [file pcbi.1006226.s003.pdf]

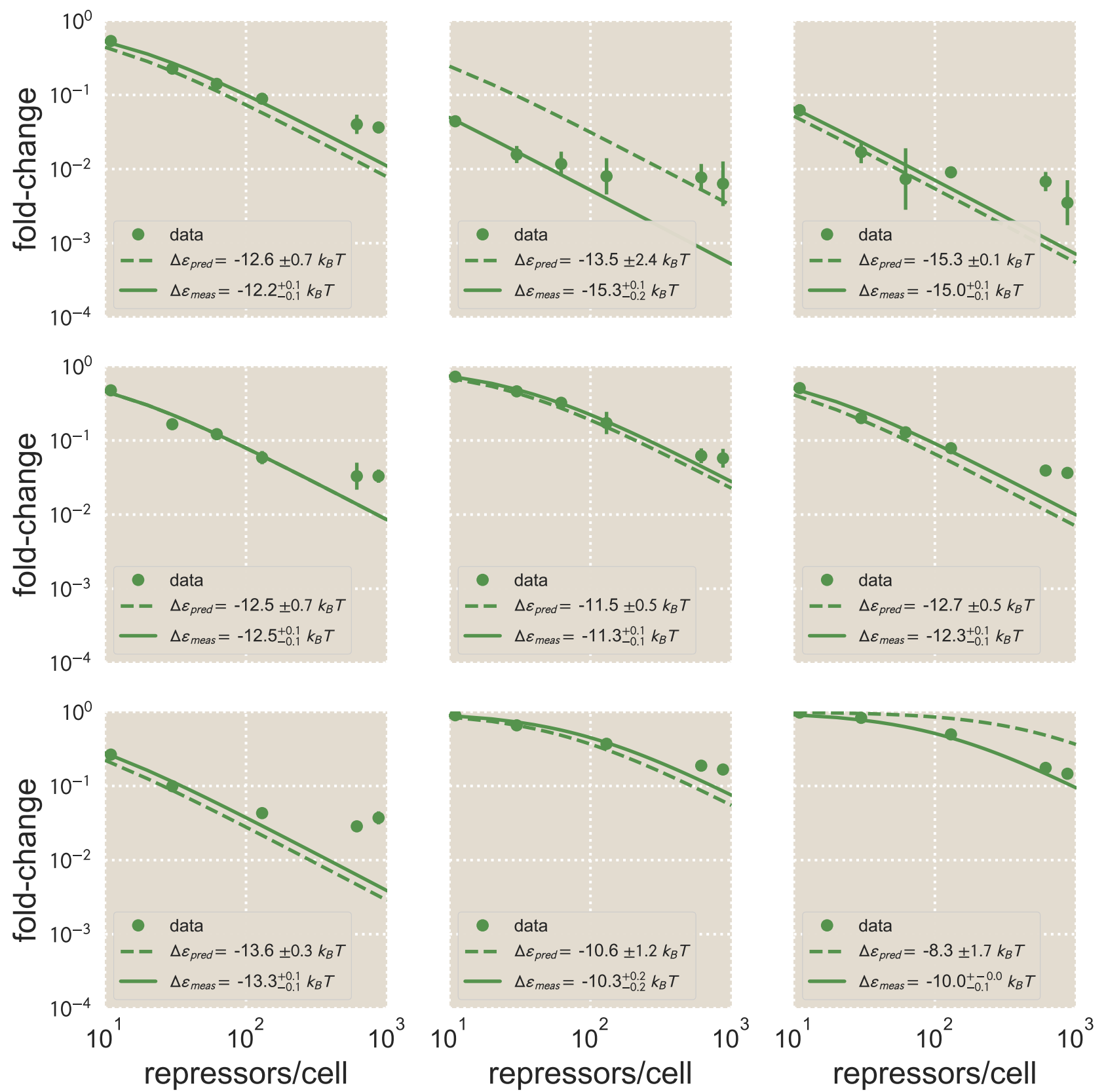

Supplement: S4 Fig — Fold-change measurements are shown for nine 1 bp operator mutants in strains with R = 11, 30, 62, 130, 610, or 870. These measurements are overlaid with the measured (fitted) binding energy measurements for each mutant and the predicted measurements as listed in the main text. Predicted energy values are listed along with the standard deviation in predictions, and measured energy values are listed along with the 95% confidence intervals for the fitted energies. Note that the bottom three plots do not display data points for R = 62, as the data for these strains were outliers. (PDF) [file pcbi.1006226.s004.pdf]

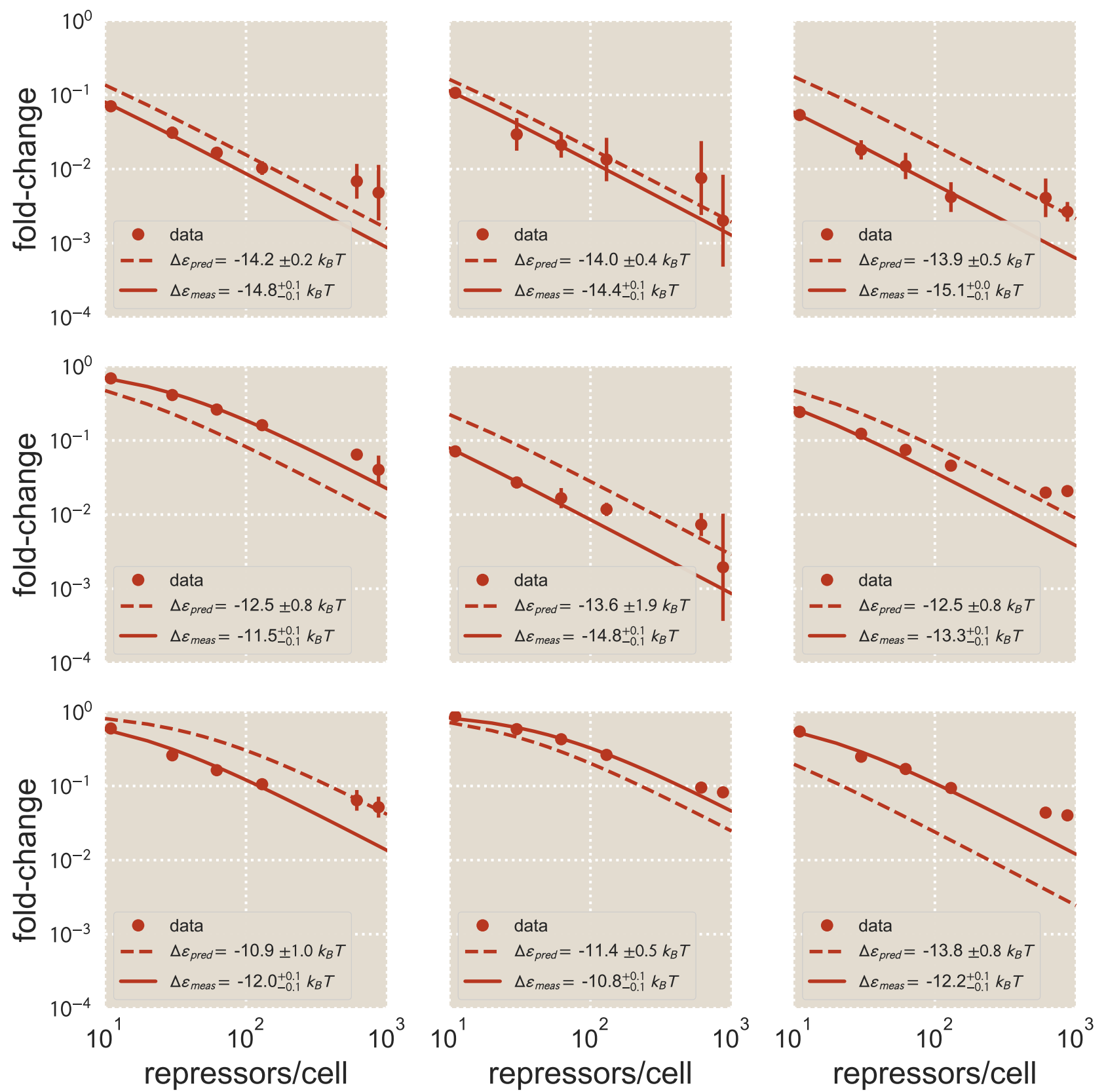

Supplement: S5 Fig — Fold-change measurements are shown for nine 2 bp operator mutants in strains with R = 11, 30, 62, 130, 610, or 870. These measurements are overlaid with the measured (fitted) binding energy measurements for each mutant and the predicted measurements as listed in the main text. Predicted energy values are listed along with the standard deviation in predictions, and measured energy values are listed along with the 95% confidence intervals for the fitted energies. (PDF) [file pcbi.1006226.s005.pdf]

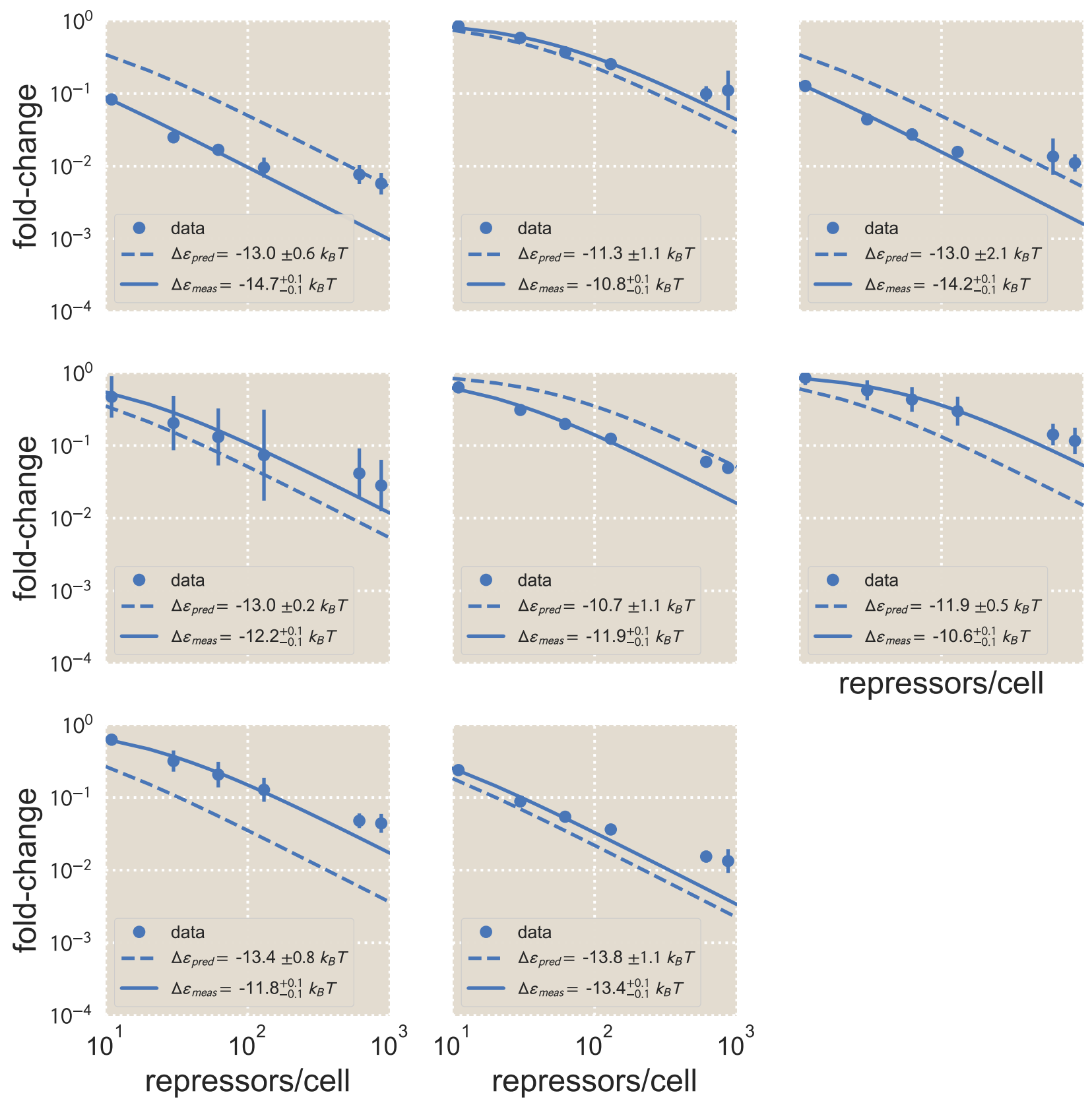

Supplement: S6 Fig — Fold-change measurements are shown for seven 3 bp operator mutants in strains with R = 11, 30, 62, 130, 610, or 870. These measurements are overlaid with the measured (fitted) binding energy measurements for each mutant and the predicted measurements as listed in the main text. Predicted energy values are listed along with the standard deviation in predictions, and measured energy values are listed along with the 95% confidence intervals for the fitted energies. (PDF) [file pcbi.1006226.s006.pdf]
