# Supplementary material for: Mapping DNA sequence to transcription factor binding energy in vivo
Source: PLoS Comput Biol. 2019 Feb 4;15(2):e1006226. doi: 10.1371/journal.pcbi.1006226 (PMC6375646; doi:10.1371/journal.pcbi.1006226)
Supplement: S1 Text — (PDF) [file pcbi.1006226.s007.pdf]

Bayesian Inference of Energy Matrix Models

We use Sort-Seq data to generate energy matrices that map sequence to binding energy. As discussed in Refs. [1, 2], one can infer these energy matrices by Bayesian parameter estimation using the observation that for large data sets,

$$p(\text{data} \mid \text{model}) \propto 2^{NI(\sigma;\mu)}, \tag{1}$$

where N is the number of data points and $I(\sigma;\mu)$ represents the mutual information between the promoter sequence σ and the fluorescence bin μ. Using a method discussed in detail in Refs. [2, 3], we use a Markov Chain Monte Carlo (MCMC) algorithm to infer a set of energy values (in arbitrary units) for each energy matrix position that maximizes the mutual information between binding site sequence and fluorescence bin. This inference is performed using the MPAthic software package [4]. In libraries where the RNAP binding sequence is also mutated, we can infer energy matrices for both the RNAP binding site and the transcription factor binding site. It is necessary to have this RNAP matrix in order to constrain the model sufficiently to infer a scaling factor for the transcription factor energy matrix, as described below. While RNAP matrices were produced as part of the inference procedure for several of the energy matrices used in this work, we do not reproduce them here as they were not directly used for any analysis.

In order to convert energy matrices into absolute energy units (such as the k_BT units used in this work), one must obtain a scaling factor that can be applied to the matrix. To obtain this scaling factor, we first observe that energy matrices derived from Sort-Seq can be used to predict the binding energy associated with a given operator mutant ($\Delta\varepsilon_R$) using the linear equation

$$\Delta\varepsilon_R = \alpha\varepsilon_{\text{mat}} + \Delta\varepsilon_{\text{wt}}, \tag{2}$$

where ε_{mat} is the energy value obtained by summing the matrix elements associated with a sequence, α is a scaling factor that converts the matrix values into k_BT units, and $\Delta\varepsilon_{\text{wt}}$ is the binding energy associated with the reference sequence. The values of the matrix positions associated with the reference sequence are fixed at 0 k_BT, so that $\varepsilon_{\text{mat}} = 0$ for the reference sequence. Thus, $\alpha\varepsilon_{\text{mat}}$ can be interpreted as the change in binding energy relative to the reference sequence caused by the specific mutations to the sequence. The value of α can be determined in a number of ways (as discussed further in S2 Text), but the method employed in the main text is to use Bayesian parameter estimation by MCMC. The advantage of this method is that if a thermodynamic model for the promoter is known, one can use the Sort-Seq data to infer the value of α without having to perform any additional experiments. Here we describe in detail how MCMC is used to infer a value for α.

If the energy matrix is properly converted into k_BT units, then one can use energy matrix predictions, along with a thermodynamic model for gene expression, to discern which fluorescence bin a given promoter sequence should have fallen into. We discuss above how one can infer the energy matrix parameters by maximizing the mutual information between sequence and expression bin. Similarly, we can obtain an estimate for α by finding the value of α that maximizes the mutual information between the Sort-Seq data and the expression predictions from the matrix and thermodynamic model. For the thermodynamic model, we begin with the expression for p_{bound} for a simple repression system,

$$p_{bound} = \frac{\frac{P}{N_{NS}} e^{-\beta\Delta\varepsilon_P}}{1 + \frac{P}{N_{NS}} e^{-\beta\Delta\varepsilon_P} + \frac{2R}{N_{NS}} e^{-\beta\Delta\varepsilon_R}}, \tag{3}$$

where P is the number of RNAP molecules in the system, N_{NS} is the number of nonspecific binding sites available in the system (i.e. the length of the genome), R is the number of repressors in the system, $\Delta\varepsilon_P$ is the binding energy of RNAP to its binding site, and $\Delta\varepsilon_R$ is the binding energy of the repressor to its binding site. We can rearrange this equation to make it easier to work with. First, we divide the top and bottom by the numerator, giving us

$$p_{bound} = \frac{1}{1 + \frac{1+\frac{2R}{N_{NS}}e^{-\beta\Delta\varepsilon_R}}{\frac{P}{N_{NS}}e^{-\beta\Delta\varepsilon_P}}}. \quad (4)$$

Importantly, in order to evaluate the mutual information between $\Delta\varepsilon_R$ and p_{bound}, it is not necessary to adhere to the full expression for p_{bound}. Rather, we can manipulate the expression in ways that make it easier for us to work with, provided that the mutual information between $\Delta\varepsilon_R$ and p_{bound} is preserved. As noted in [5], the mutual information is preserved provided that any manipulations to the expression do not disrupt the rank ordering of an expression's values as the value of $\Delta\varepsilon_R$ is varied. We note that the term $\frac{1+\frac{2R}{N_{NS}}e^{-\beta\Delta\varepsilon_R}}{\frac{P}{N_{NS}}e^{-\beta\Delta\varepsilon_P}}$ has the same rank ordering as the full expression for p_{bound}. Furthermore, taking the log of this term will also not affect the rank ordering, and it will make the calculation simpler, so we take the log to get an expression which we will refer to as p'_{bound}, giving us

$$p'_{bound} = \ln\left(1 + \frac{2R}{N_{NS}}e^{-\beta\Delta\varepsilon_R}\right) - \ln\left(\frac{P}{N_{NS}}\right) + \beta\Delta\varepsilon_P. \quad (5)$$

We observe that the constant $\ln\left(\frac{P}{N_{NS}}\right)$ also does not affect rank ordering, so we can drop this term. Additionally, we recall that $\Delta\varepsilon_R = \alpha\epsilon_{mat,R} + \Delta\varepsilon_{wt,R}$. Likewise, we can say that $\Delta\varepsilon_P = \gamma\epsilon_{mat,P} + \Delta\varepsilon_{wt,P}$, where γ is the scaling factor for the RNAP matrix. As before, we can drop the constant $\Delta\varepsilon_{wt,P}$ as it will not affect rank ordering. This leaves us with the expression

$$p'_{bound} = \ln\left(1 + \frac{2R}{N_{NS}}e^{-\beta(\alpha\varepsilon_{mat,R} - \Delta\varepsilon_{wt,R})}\right) + \beta\gamma\varepsilon_{mat,P}. \quad (6)$$

With this expression in hand we can sample values of γ and α to identify values that maximize the mutual information between p'_{bound} and the expression bin which a particular sequence was sorted into during Sort-Seq. Note that while the rest of the discussion will focus on α, a value for γ comes out of this analysis as well.

The mutual information surface is very rough, with many peaks, so we need to use a method which can avoid getting stuck in local maxima. We use a parallel tempering MCMC algorithm to achieve this [6]. The parallel tempering MCMC algorithm works by randomly sampling possible values for α and rejecting the value with some probability if it does not increase the mutual information relative to the previous sampled value of α. In this respect it is similar to a "standard" MCMC algorithm. By contrast with a standard MCMC algorithm, a parallel tempering algorithm runs multiple chains at once at different temperatures. In our case, we use 10 different temperatures ranging from $\beta = 0.02$ to $\beta = 4$ on a log scale, where $\beta = 1/k_BT$. Periodically throughout the MCMC run, the current α values from different temperature chains will swap. This allows the algorithm to sample α values at different levels of precision. Specifically, the high temperature chains will explore widely and not get stuck in local minima, while the low temperature chains will then carefully explore the peak that was found by the high temperature chain. The output is a distribution of values, and we take the median of this distribution to obtain our estimate for α.

To quantify the error associated with our energy matrices, we elected to compare replicate energy matrices inferred using the methods described above. As discussed in S4 Text, we performed three biological replicates each for the energy matrices with O1 reference sequence and repressor copy number $R = 30$, O1 reference sequence and $R = 62$, O2 reference sequence and $R = 30$, and O2 reference sequence and $R = 62$. It was not feasible to perform biological replicates for every matrix studied in this work, so in several cases we instead used a technique we refer to here as "sequence-splitting," in which a sequencing data set is split into three separate groups of nonoverlapping sequences which can be assumed to be independent. Each of these groups of sequences is used to produce an energy matrix as described above. To evaluate whether this provides similar statistics to the biological replicates, in Figure 1 we compare the energy matrix values derived from each method for energy matrices obtained using an O1 reference sequence and $R = 62$. For the sequence-splitting data we used the "Day 1" Sort-Seq data. In Figure 1A we directly plot the values from each resulting energy matrix against one another. We see

that the mean energy matrix values agree overall with one another ($r = 0.97$). To determine whether the methods infer a similar amount of error, in Figure 1B we display box plots overlaid with beeswarm plots of the coefficient of variation (CV) associated with each data point for each inference method. We find that the distribution of CVs for each method is quite similar, with a median CV of 0.11 for the sequence-splitting method and a median CV of 0.12 for the multi-day replicate method. Thus, we conclude that for the case of matrices made using an O1 reference sequence, sequence-splitting replicates give similar statistics to multi-day biological replicates.

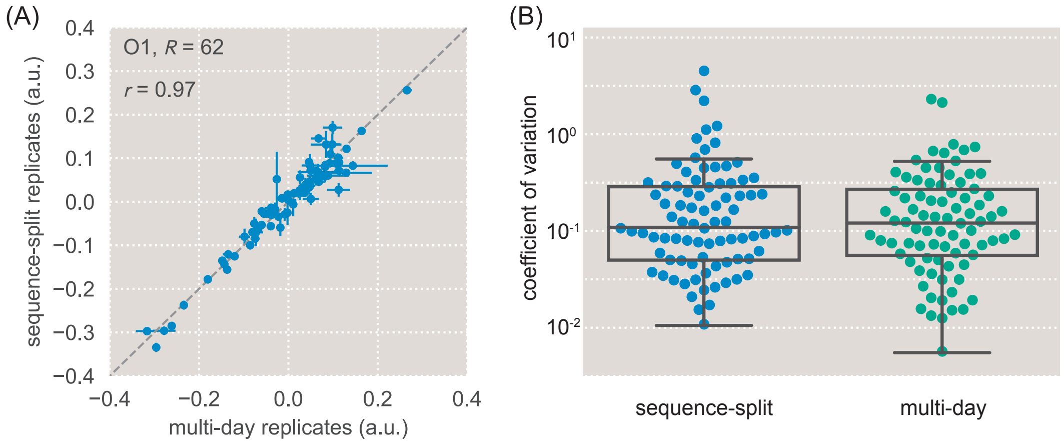

Figure 1. Comparison of alternate methods of obtaining energy matrix error bars. (A) We infer mean energy matrix values and standard deviation error bars using two different methods for Sort-Seq experiments using an O1 reference sequence and $R = 62$. The first method (multi-day replicates, x-axis) uses data from three separate biological replicates taken on three different days. The second method (sequence-splitting, y-axis) uses a single biological replicate, but splits the sequence data into three groups of sequences and treats each group as a separate replicate. (B) The range in CV is shown for the sequence-splitting replicates (blue) and the multi-day replicates (green). A box-plot is overlaid for each set of replicates.

References

1. Kinney JB, Tkačik G, Callan CG. Precise physical models of protein-DNA interaction from high-throughput data. Proceedings of the National Academy of Sciences. 2007;104(2):501–506.

2. Kinney JB, Murugan A, Callan CG, Cox EC. Using deep sequencing to characterize the biophysical mechanism of a transcriptional regulatory sequence. Proceedings of the National Academy of Sciences. 2010;107(20):9158–9163.

3. Atwal GS, Kinney JB. Learning quantitative sequence–function relationships from massively parallel experiments. Journal of Statistical Physics. 2016;162(5):1203–1243.

4. Ireland WT, Kinney JB. MPAthic: quantitative modeling of sequence-function relationships for massively parallel assays. bioRxiv. 2016;.

5. Kinney J, Atwal GS. Parametric inference in the large data limit using maximally informative models. Neural Computation. 2014;26(4):637–653.

6. Earl DJ, Deem MW. Parallel tempering: Theory, applications, and new perspectives. Physical Chemistry Chemical Physics. 2005;7:3910–3916.
