# Supplementary material for: Mapping DNA sequence to transcription factor binding energy in vivo
Source: PLoS Comput Biol. 2019 Feb 4;15(2):e1006226. doi: 10.1371/journal.pcbi.1006226 (PMC6375646; doi:10.1371/journal.pcbi.1006226)
Supplement: S2 Text — (PDF) [file pcbi.1006226.s008.pdf]

Alternate Methods for Obtaining Energy Matrix Scaling Factor

As discussed in S1 Text, in order to convert an energy matrix into k_BT units one must infer an appropriate scaling factor α. In the main text we primarily use Bayesian parameter estimation by MCMC to infer this factor, but other methods can be used as well. Here we discuss two alternative methods: least squares regression to measured binding energy values, and calibrating to a theoretical mutation parameter. In this Text we will discuss the strengths and weaknesses of each method and compare predictions using these methods to predictions using MCMC.

Fitting by Least Squares Regression to Measured Binding Energy Values

To obtain a value for α using least squares regression, we first define a least-squares function $f(\alpha)$ as

$$f(\alpha) = \sum_{i=1}^{n} \left(\Delta\varepsilon_{meas,i} - \alpha\Delta\varepsilon_{pred,i} - \Delta\varepsilon_{wt}\right)^2, \tag{1}$$

where $\Delta\varepsilon_{meas}$ is the measured binding energy for an operator mutant, $\Delta\varepsilon_{pred}$ is the corresponding binding energy prediction from our unscaled energy matrix, and $\Delta\varepsilon_{wt}$ is the binding energy of the reference sequence used to generate the matrix. To determine the best-fit value of α, we identify the value of α that minimizes the function. We perform this fit using measurements from the nine single base pair mutants used in this work.

Fitting to the Average Energy per Mutation

In many cases, we will not have thermodynamic models available to use for inferring scaling factors by fitting or Bayesian inference. This raises the question of whether it is possible to estimate the scaling factor by other means, for example by determining some average binding penalty incurred by making a mutation to a binding site. To explore how we might think about such an average binding penalty, we consider the effects of mutations away from the lowest-energy binding sequence for LacI (Figure 1), as derived from the mean energy matrix produced with reference sequence O1 and $R = 130$. As shown in Figure 1A, a wide range of binding energies are available to binding site mutants. The distribution of binding penalties of single base-pair mutations to this binding site is shown in Figure 1B. The distribution is fairly broad, yet we find that the mean predicted binding energy for binding site mutants, as shown in Figure 1C, is strongly related to the mean binding penalty of a single base pair mutation. Specifically, the slope of the predicted energy versus the number of mutations is approximately equal to the mean binding penalty of a single mutation. This tells us that the average energy per mutation is a meaningful metric that provides information about the general behavior of a transcription factor binding site.

Next we need to determine how one would estimate the average energy per mutation for an energy matrix that has not already been converted into absolute energy units. We turn to Ref. [1] in which they make an estimate for the average energy penalty, ε_{mut}, of a single base pair mutation relative to the minimum-energy sequence. We can use this estimate to infer a value for α when no thermodynamic model is available to perform a fit for α. We note that unlike the other methods for obtaining α, this method does not rely on expression information from the promoter of interest and thus is best interpreted as a "rough guess."

To begin this estimate, we assume a minimal organism in which there is a single transcription factor with a copy number of 1, and this transcription factor regulates gene expression by binding to a single minimum-energy operator, which has an energy of $\Delta\varepsilon_{min}$. The remaining sequence in this minimal genome is mostly random, but it includes a number of weaker binding sites for the transcription factor such that all possible single base-pair mutations to the binding site are represented. From a statistical mechanics perspective, in order for the transcription factor to bind reliably to the minimum-energy operator, the operator's statistical weight (given by $e^{-\beta\Delta\varepsilon_{min}}$) must outweigh the total statistical weight of all possible single base-pair binding site mutants (given by $le^{-\beta(\Delta\varepsilon_{min}+\varepsilon_{mut})}$), where l is the length of the binding site in base pairs. This gives us

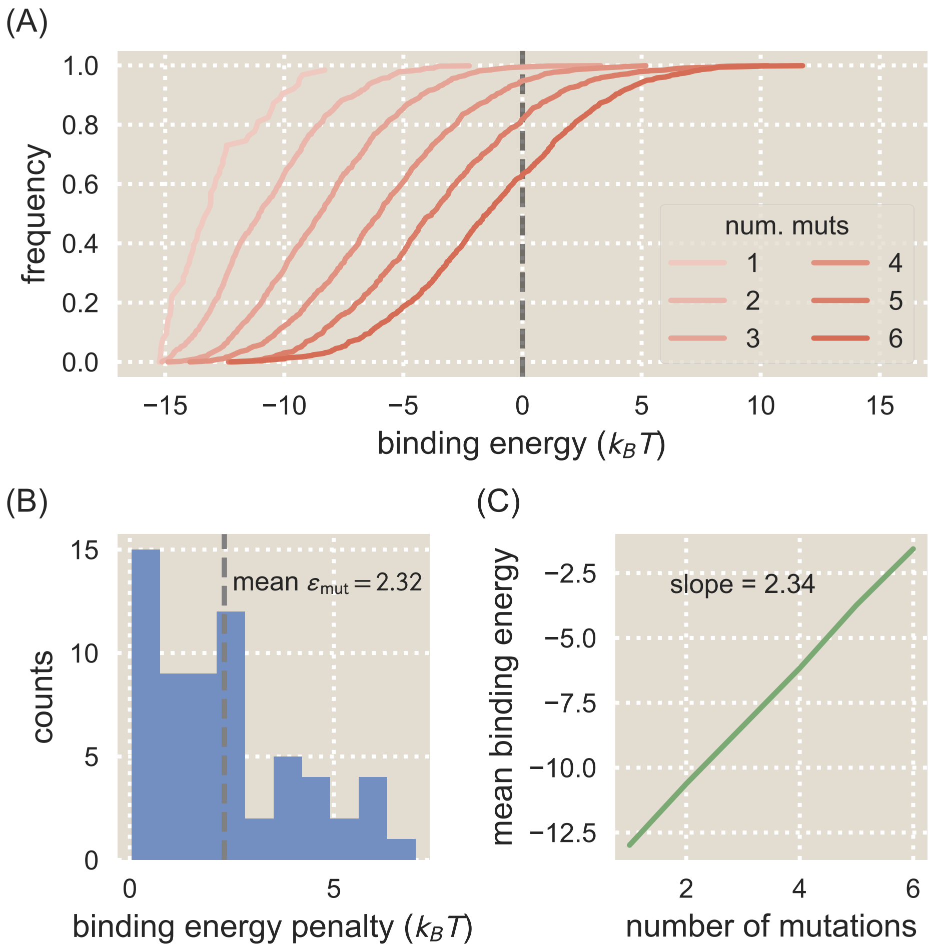

Figure 1. Average effect of a binding site mutation. (A) Cumulative distributions are shown for the predicted binding energies of *lac* operator mutants. The mean predicted binding energy increases substantially with the number of mutations, as does the width of the distribution. The dotted line shows the point at which $\Delta\varepsilon_R = 0\ k_BT$, which is the average energy of nonspecific binding. (B) A histogram of binding penalties for single base-pair mutations to the minimum-energy LacI binding sequence shows that the mean binding penalty of a mutation is 2.32 k_BT. (C) Plotting the mean binding energy of an operator against the number of mutations relative to the minimum-energy sequence shows a linear trend with a slope approximately equal to the average energy penalty per mutation.

$$e^{-\beta\Delta\varepsilon_{min}} \geq le^{-\beta(\Delta\varepsilon_{min}+\varepsilon_{mut})}. \tag{2}$$

This implies that the minimum average binding energy penalty due to a mutation is given by $\varepsilon_{mut} = \ln l$, which for a binding site of 21 bp (the length of a *lac* operator) comes out to $\varepsilon_{mut} \approx 3\ k_BT$. This is similar to the mean energy penalty of 2.32 k_BT calculated for LacI as noted in Figure 1B. In this work, we typically use a value of 2.5 k_BT, which accounts for the fact that some bases in a binding site contribute minimally to binding, and serves as an intermediate estimate that can be used for binding sites of varying length.

Based on this estimate, one can find a value for α by setting the minimum binding energy of an energy matrix to 0, then taking the mean of the nonzero elements of the matrix, ε_{mean}, and finding a scaling factor α such that $\alpha\varepsilon_{mean} = \ln l k_BT$.

Method comparison

Each of the methods outlined above is capable of producing a value of α that can be used to convert an energy matrix into $k_B T$ units. Each of these methods has its own advantages and disadvantages. Here we will outline these trade-offs and compare the accuracy of the predictions that can be made using each method.

The primary advantage of the Bayesian regression by MCMC method, which is used for the LacI binding energy predictions in the main text, is that it can be implemented using the same Sort-Seq data that was used to obtain the energy matrices. No further data collection is required. However, in order to implement this method one must have a thermodynamic model that predicts gene expression for a given operator binding energy. This is trivial for systems with simple regulatory architectures, as is the case with the simple repression architecture used in this study. However, while models for more complex architectures have been proposed [2], identifying the correct model may not be straightforward and a number of additional experiments may be required in order to validate the proposed model. Additionally, significant computing power is required in order to infer a scaling factor using this method.

The advantages of the least-squares fitting method are that it is conceptually straightforward, it requires little computing power, and it provides a very accurate scaling factor. However, multiple fold-change measurements for different operator mutants are required to perform the regression and calculate the best-fit value of α, and any outliers must be identified in order to maximize the accuracy of the fit. Additionally, a thermodynamic model for the system is again required if binding energies are to be measured using fold-change data.

The advantage of the theoretical mutation parameter method is that it is very simple and requires no knowledge of the regulatory architecture of the promoter. All that is needed is an energy matrix for an operator and an estimate of the operator's length. Indeed, for XylR we lack sufficient information to confidently infer a thermodynamic model of gene expression, so this is the method used to produce energy matrices for this transcription factor (we note that the theoretical mutation parameter method is also used for PurR energy matrices in the main text, though a thermodynamic model is available for PurR [3]). For the *lac* operator it produces a scaling factor that is approximately as accurate as the other inference methods discussed here (see Figure 2). However, this method is based on simplified biophysical arguments, and it is likely that there are a number of regulatory scenarios for which it would not be as successful.

Figure 2 compares predictions made using each method for obtaining a scaling factor. The same Sort-Seq data set was used for each prediction, with O1 as the wild-type sequence and $R = 130$ LacI tetramers in the strain used for Sort-Seq. As in the main text, we split the data set into three groups of sequences and create an energy matrix for each, which we use to obtain replicate predictions for each mutant operator. We find that all methods produce predictions that generally describe the data, but when comparing the mean squared error (MSE) of the mean values of the predictions, it is clear that some methods perform better than others. Note that elsewhere in the supplement we compare predictions using the Pearson correlation coefficient (r). We use the MSE here instead because an inaccurate scaling factor will not affect the linear relationship between predictions and measurements, but it will affect the accuracy of the predictions. Thus a set of predictions may have a high r value corresponding to a strong linear relationship, but still have a high MSE corresponding to inaccurate predictions. The Bayesian parameter estimation (Figure 2A) and least-squares regression (Figure 2B) methods perform nearly identically. However, while the value for α that was inferred from the theoretical mutation parameter (Figure 2C) makes predictions that generally describe the data, the MSE values associated with its predictions are notably larger than the other methods, particularly for the 1 bp mutants. It is also notable that the size of the error bars associated with each method varies considerably. Each of the plots in Figure 2 was generated using the same set of three matrix replicates from a single Sort-Seq experiment, yet the error bars associated with the least-squares method are considerably smaller than either of the other methods. In the case of the Bayesian parameter estimation method, this is most likely due to error inherent in estimating the scaling factor for each replicate matrix from MCMC, which magnifies the variation associated with each replicate matrix.

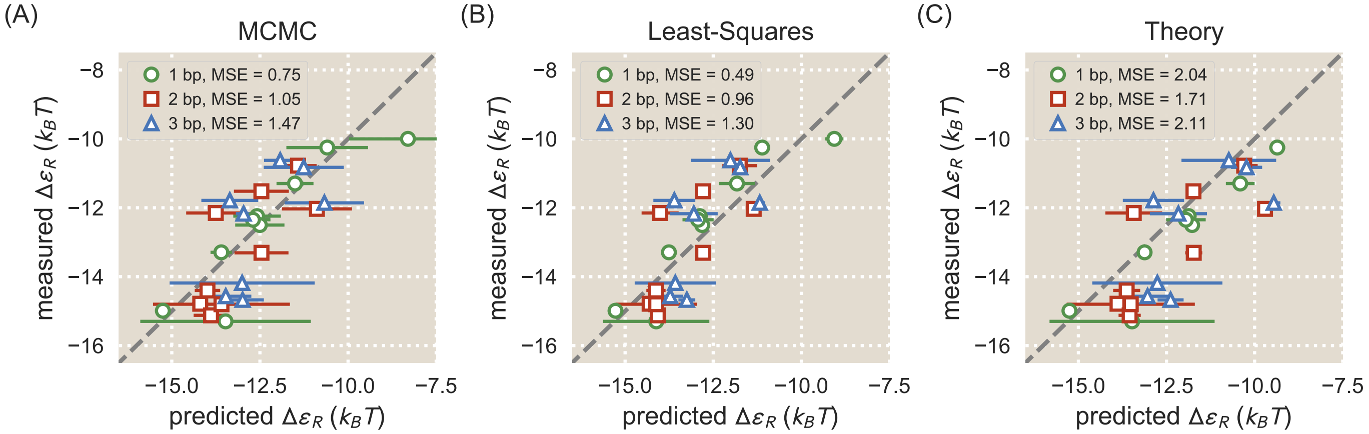

Figure 2. Alternate methods of obtaining energy matrix scaling factor produce similar results. Shown are data for predicted vs. measured binding energies of 1, 2, or 3 bp mutants, predicted using energy matrices with reference sequence O1 and $R = 130$. The binding energy predictions are made using energy matrices that have been scaled using one of three methods: (A) Bayesian parameter estimation using MCMC, (B) least-squares regression, or (C) inference from a theoretical mutation parameter. All predictions were made using an energy matrix with O1 as the reference sequence and $R = 130$ LacI tetramers in the cells used to perform Sort-Seq. The mean squared error (MSE) associated with each set of predictions is noted in the legend. Error bars represent the standard deviation of binding energy predictions using replicate matrices from the same Sort-Seq data set.

References

1. Lässig M. From biophysics to evolutionary genetics: statistical aspects of gene regulation. BMC Bioinformatics. 2007;8(Suppl 6):S7.

2. Bintu L, Buchler NE, Garcia HG, Gerland U, Hwa T, Kondev J, et al. Transcriptional regulation by the numbers: models. Current Opinion in Genetics and Development. 2005;15:116–124.

3. Belliveau NM, Barnes SL, Ireland WT, Jones DL, Sweredoski M, Moradian A, et al. A systematic approach for dissecting the molecular mechanisms of transcriptional regulation in bacteria. Proceedings of the National Academy of Sciences. 2018;115.
