# Supplementary material for: Mapping DNA sequence to transcription factor binding energy in vivo
Source: PLoS Comput Biol. 2019 Feb 4;15(2):e1006226. doi: 10.1371/journal.pcbi.1006226 (PMC6375646; doi:10.1371/journal.pcbi.1006226)
Supplement: S3 Text — (PDF) [file pcbi.1006226.s009.pdf]

Comparing single-point energy matrix models with higher-order models

A commonly cited problem with the type of energy matrices used in this work is that they do not accurately describe the mechanism of transcription factor binding to DNA. While such energy matrix models assert that each base pair contributes independently to the binding energy, it is known that interactions between two or more base pairs can play an important role in determining binding affinity [1,2]. In spite of this, energy matrix models that assume independence (which we will refer to here as single-point models) are still commonly used because they often perform nearly as well as higher-order models [3,4], and they require many fewer parameters than a higher-order model. For example, a single-point model for LacI binding to a 21 bp long operator requires that 84 parameters be inferred, one for each base at each position. By contrast, a two-point model for LacI that accounts for all possible interactions between any two bases in the binding site requires 3660 parameters. Obtaining high-quality estimates for these parameters requires a great deal more data and computing power than inferring parameters for single-point models. Thus it is important to carefully consider whether higher-order models will dramatically improve predictions.

Here we take advantage of our large Sort-Seq data sets to infer two-point binding energy models for LacI binding. As with single-point models, two-point binding energy models are inferred by identifying a set of parameters that maximizes mutual information between sequence and expression bin (see S1 Text for more details). In Figure 1 we compare binding energy measurements to predictions from a single-point model (Fig. 1(A)) and a two-point model (Fig. 1(B)). Unlike in the main text, here we do not split the Sort-Seq data into replicate data sets. This allows us to use the full array of sequences to infer matrix parameters for all possible two-point interactions. We also make this comparison for models in which sequences with only one sequencing count are removed from the data set and then all other sequences are weighted equally 1(C-D)). This weighting scheme removes possible sequencing errors from the data set and then gives low-frequency sequences the same influence as high-frequency sequences, compensating for any inequalities that may arise if the library itself has an unequal representation of sequences. The same data set was used to infer each model, namely the data set for the strain with repressor copy number $R = 130$ and an O1 reference sequence. The quality of the predictions for each model is quantified by noting the Pearson's correlation coefficient r for each data set. Surprisingly, the unweighted two-point model does not outperform the single-point model. In fact, it performs substantially worse. The weighted two-point model, however, performs better than the weighted single-point model.

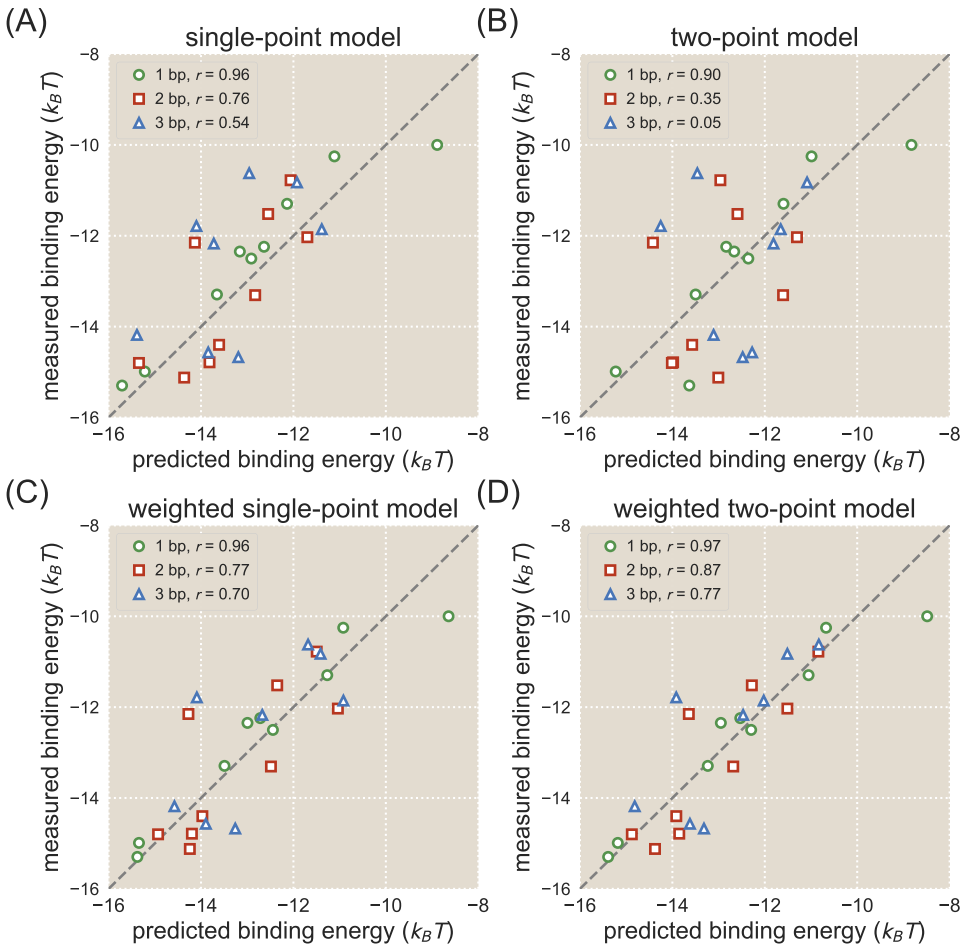

Figure 1. A comparison of single-point models with two-point models. Binding energy measurements are compared against predictions from energy matrix models obtained using a strain where $R = 130$ and O1 is the reference sequence. (A) Predictions are made using a single-point energy matrix in which each sequence position is considered independently. This matrix is used to obtain the predictions discussed in the main text. (B) Predictions are made using an energy matrix model that accounts for all two-point interactions between nucleotides at different sequence positions. The Pearson's correlation coefficients for the measurements and predictions indicate that this matrix model performs substantially worse than the single-point energy matrix model, particularly for multiple mutations. (C) Predictions are again made using a single-point energy matrix model, though this model has been weighted so that all sequences (aside from single-count sequences, which were dropped) have the same weight. This matrix model has been inferred after removing all single-count sequences from the data set and then weighting all sequences evenly. (D) Predictions are made using a two-point matrix model using the same weighting scheme as in (C). This weighting procedure results in a two-point matrix model that makes improved predictions relative to the weighted single-point energy matrix model.

References

1. Benos PV, Bulyk ML, Stormo GD. Additivity in protein-DNA interactions: how good an approximation is it? Nucleic Acids Research. 2002;30(20):4442–4451.

2. Siebert M, Söding J. Bayesian Markov models consistently outperform PWMs at predicting motifs in nucleotide sequences. Nucleic Acids Research. 2016;44(13):6055–6069.

3. Zhao Y, Stormo GD. Quantitative analysis demonstrates most transcription factors require only simple models of specificity. Nature Biotechnology. 2011;29(6):480–483.

4. Weirauch MT, Cote A, Norel R, Annala M, Zhao Y, Riley TR, et al. Evaluation of methods for modeling transcription factor sequence specificity. Nature Biotechnology. 2013;31(2):126–134.
