# Supplementary material for: Mapping DNA sequence to transcription factor binding energy in vivo
Source: PLoS Comput Biol. 2019 Feb 4;15(2):e1006226. doi: 10.1371/journal.pcbi.1006226 (PMC6375646; doi:10.1371/journal.pcbi.1006226)
Supplement: S4 Text — (PDF) [file pcbi.1006226.s010.pdf]

Text

Influence of Regulatory Parameters on Energy Matrix Quality

The level of repression in a repressible system is dependent on a number of factors. In this work we primarily focus on operator binding energy, but other key parameters include operator copy number, repressor copy number, and competition from other binding sites, as discussed in detail in Ref. [1]. Here we consider how two parameters influence energy matrix quality: namely, repressor copy number R and the binding energy of the operator reference sequence.

Because our promoter constructs are on plasmids and thus have multiple copies ($N \approx 10$), there is some concern that there might not be a sufficient number of repressors in the cell to demonstrate significant changes in expression when the *lac* operator is mutated. The wild-type copy number of LacI tetramers in *E. coli* is $R = 11$, which is comparable to the plasmid copy number used in this study. We increase the LacI copy number by using synthetic RBSs that have been shown to increase gene expression [2]. Additionally, we consider the fact that the binding energy of the reference sequence influences the distribution of binding energies present in the mutant library, and therefore the "ideal" value of R may be different for different reference sequences. To explore these factors, we performed Sort-Seq experiments for each combination of R (i.e. $R = 30, 62, 130$, or 610) and reference binding energy (i.e. $\Delta\varepsilon_{\mathrm{R}} = -15.3\ k_BT$ for O1, $\Delta\varepsilon_{\mathrm{R}} = -13.9\ k_BT$ for O2, or $\Delta\varepsilon_{\mathrm{R}} = -9.7\ k_BT$ for O3).

Comparison of binding energy predictions

Figure 1 shows how predicted and measured binding energy values for single base pair mutants compare for each combination of repressor copy number and reference sequence. As in the main text, each Sort-Seq data set is split into three replicate data sets, each of which is used to create a predictive energy matrix. We show predictions from energy matrices that have been scaled using the least squares method (see S2 Text), as this is the most accurate method for obtaining a scaling factor. The Pearson's correlation coefficient (r) for the means from each set of predictions is shown as a way of quantifying which of these combinations produces the "best" energy matrices, as defined by which matrices give the best agreement between prediction and measurement. We see that the best agreement between prediction and measurement occurs when O1 is the reference sequence. Conversely, predictions from matrices made using O3 as a reference sequence do not predict the measured values at all, as indicated by the especially low r values. While the choice of repressor copy number does not appear to have a large effect on the quality of matrix predictions, particularly for matrices with O1 as the reference sequence, we do observe that $R = 610$ consistently corresponds with the most accurate predictions. We note that in the main text we make predictions using the energy matrix with the O1 reference sequence and $R = 130$. This is because in the main text we obtain our scaling factors using Bayesian inference by MCMC (see S1 Appendix), and the most accurate scaling factor inferred by this method was for $R = 130$.

Variation in energy matrix replicates

We performed a number of biological replicates using both O1 and O2 reference sequences to determine the level of variation in sequence logos. Sort-Seq was performed on a different day for each replicate. For these replicates, we did not split each Sort-Seq data set into multiple groups as is typically done in the main text, but rather used all of the data from a particular Sort-Seq experiment to infer the energy matrix for that experiment. As shown in Figure 2, replicates using O1 as a reference sequence produce very consistent sequence logos, while replicates using O2 as a reference sequence produce less consistent sequence logos. This suggests that the strength of the binding site is a significant factor determining the consistency of experiment outcomes.

As another point of comparison, we compared the values making up our energy matrices against one another to assess their consistency. Specifically, we computed the Pearson's correlation coefficient r between the lists of values comprising each of our unscaled energy matrices with O1 and O2 reference sequences (see Figure 3). In addition to the matrices analyzed in Figure 1, we performed two additional replicates for each of the energy matrices obtained from strains with $R = 30$ or $R = 62$. This allows us

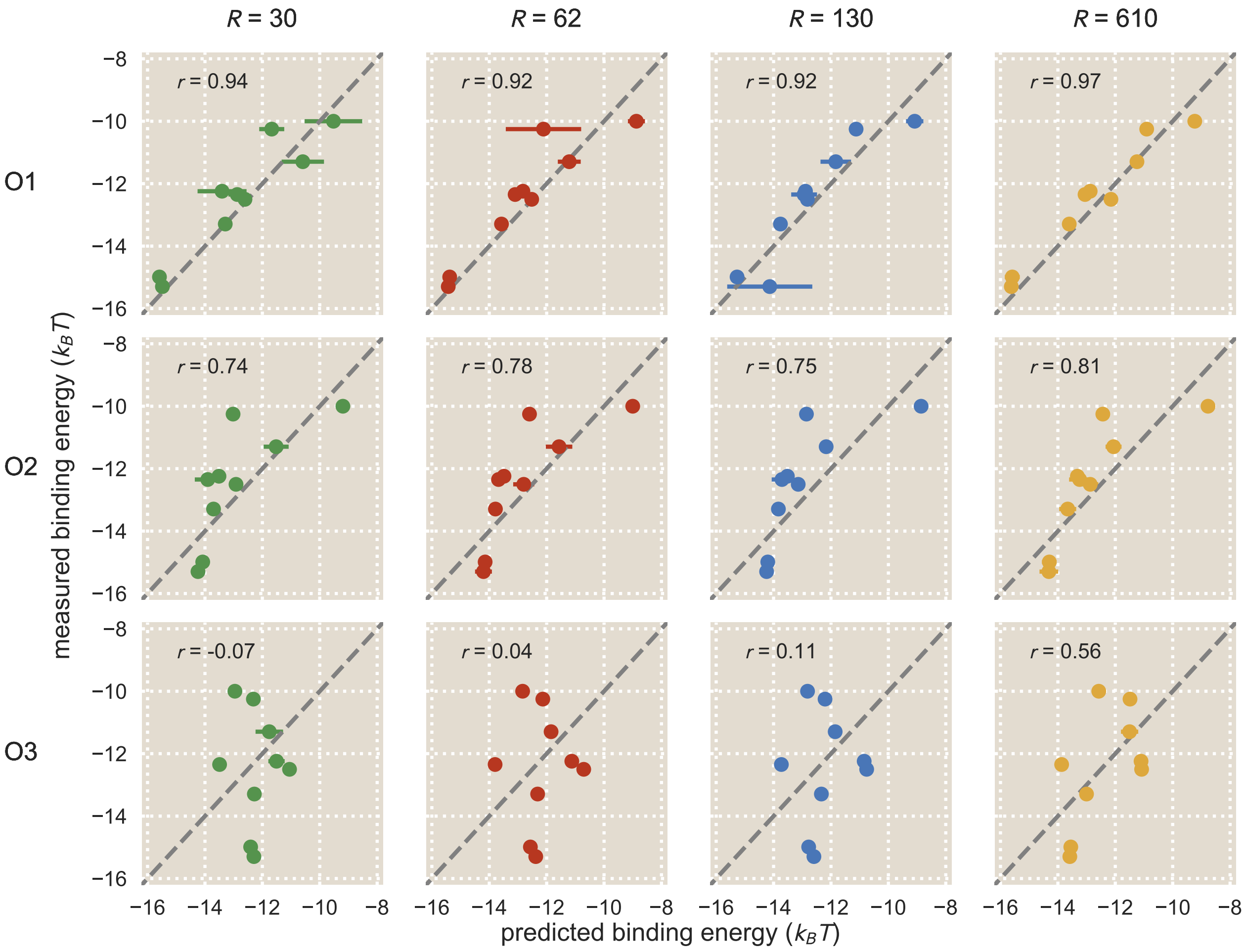

Figure 1. Repressor copy number and reference sequence affect accuracy of energy matrix predictions. Sort-Seq was performed with all combinations of four different repressor copy numbers ($R = 30, 62, 130$, and 610) and three different reference operator sequences (O1, O2, and O3) to produce a total of 12 energy matrices. Predictions from each of these energy matrices are plotted against measured binding energy values for nine single base-pair mutants. The Pearson's correlation coefficient (r) is noted for each plot as a measure of prediction accuracy. Error bars represent the standard deviation of predictions made from three replicate energy matrices for each combination of reference sequence and repressor copy number.

to ascertain whether the matrices themselves are substantially different under different experimental conditions.

We find that all of the matrices with an O1 reference sequence are highly correlated with one another. By contrast, the matrices with an O2 reference sequence are less correlated with one another, even among replicates of the same experimental conditions. The second replicate of the O2 matrix with $R = 30$ is particularly poorly correlated with other matrices. However, the O2 matrices do generally have a higher r value with one another than with the O1 matrices. An exception to this is the O2 matrices with $R = 130$ and $R = 610$, which appear to be moderately well-correlated with the O1 matrices. These results suggest that the choice of reference sequence used to perform the Sort-Seq experiment is a more important determinant of matrix quality than repressor copy number, though the results may also support the hypothesis that higher repressor copy numbers correspond with improved matrix quality, particularly for weaker reference sequences such as O2.

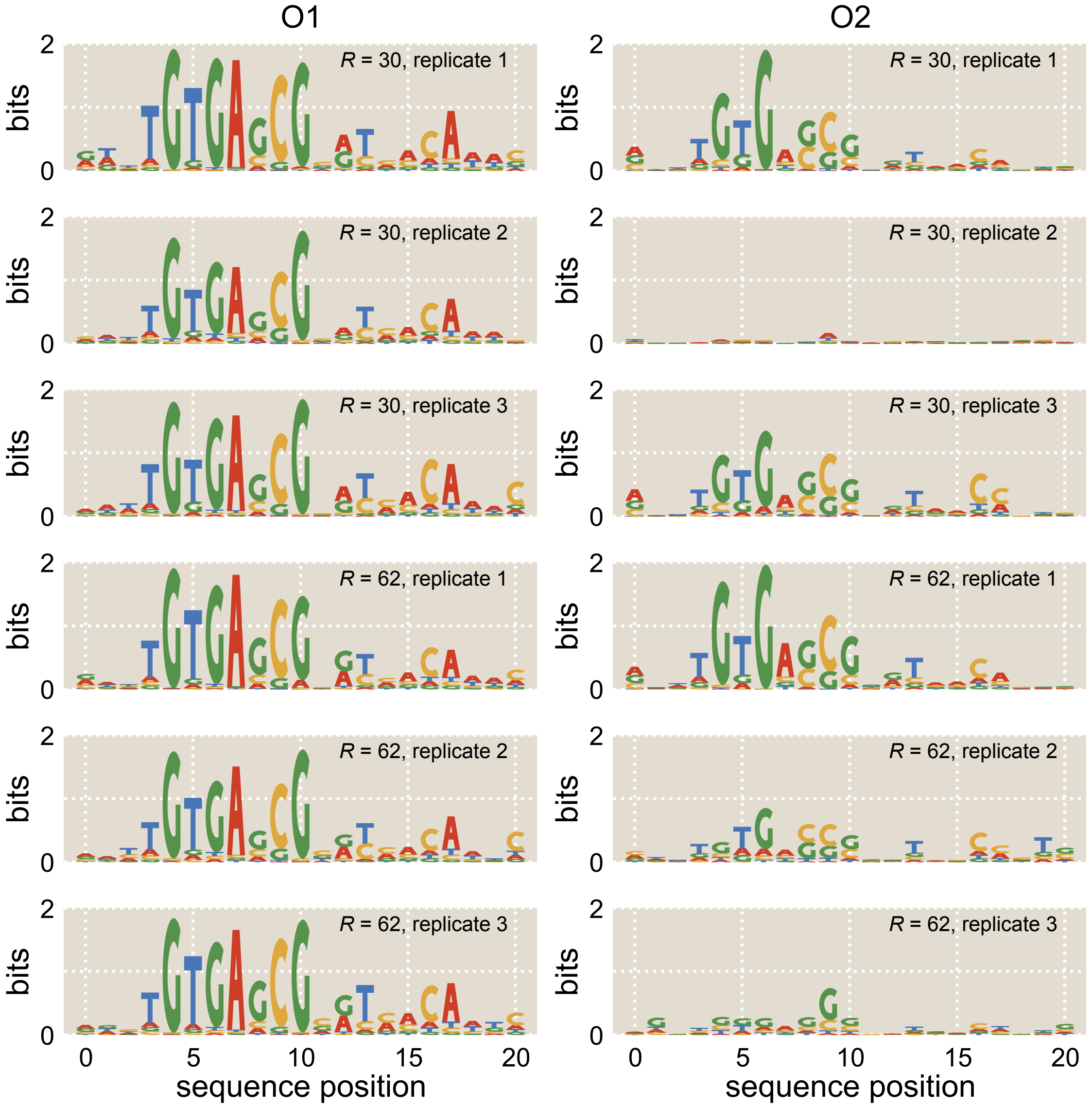

Figure 2. Variation in sequence logo results. Replicates of Sort-Seq experiments were performed using O1 or O2 as a reference sequence. The O1 experiments (left) produced very consistent sequence logos, while the O2 experiments (right) produced sequence logos that varied significantly in quality.

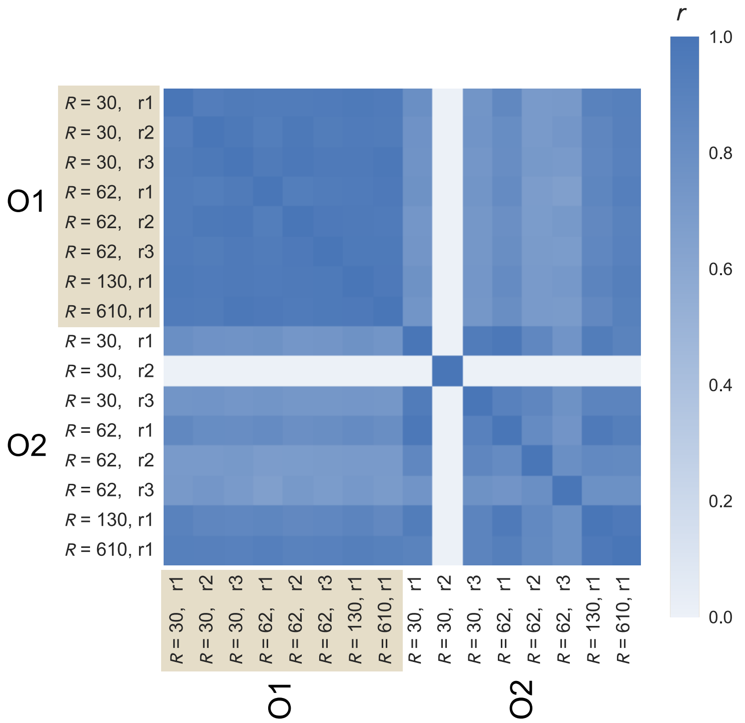

Figure 3. Correlation coefficients between unscaled energy matrices. The Pearson's correlation coefficient (r) was calculated for each pair of energy matrices with an O1 or O2 reference sequence. Those experiments conducted using strains with repressor copy number $R = 30$ and $R = 62$ were repeated three times, as denoted by replicate number r1, r2, or r3. We find that all O1 matrices are highly correlated with one another, while O2 matrices are generally less correlated with one another. In general there is low correlation between O1 and O2 matrices, with the exception of O2 matrices with high repressor copy numbers, $R = 130$ and $R = 610$.

References

1. Weinert FM, Brewster RC, Rydenfelt M, Phillips R, Kegel WK. Scaling of gene expression with transcription-factor fugacity. Physical Review Letters. 2014;113(25):1–5. doi:10.1103/PhysRevLett.113.258101.

2. Garcia HG, Phillips R. Quantitative dissection of the simple repression input-output function. Proceedings of the National Academy of Sciences. 2011;108(29):12173–12178.
