# Supplementary material for: Mapping DNA sequence to transcription factor binding energy in vivo
Source: PLoS Comput Biol. 2019 Feb 4;15(2):e1006226. doi: 10.1371/journal.pcbi.1006226 (PMC6375646; doi:10.1371/journal.pcbi.1006226)
Supplement: S5 Text — (PDF) [file pcbi.1006226.s011.pdf]

Comparison of full-promoter and operator-only energy matrix predictions

In the main text, we perform Sort-Seq using libraries in which the entire promoter region was mutated, namely both the RNAP site and the operator. Here we consider whether one can improve energy matrix accuracy by using libraries in which only the operator is mutated.

In order to infer the energy matrix scaling factor α from Sort-Seq data alone (see S1 Text), it is necessary to mutate the full promoter, because mutations to both the operator and RNAP binding sites are relevant to the thermodynamic model used to perform the inference. Because of this we use full promoter mutant libraries in the main text. This means that an alternate method is required in order to infer an energy matrix scaling factor for matrices derived from libraries in which only the operator was mutated. Here, we obtain a scaling factor by least-squares regression to a set of measured binding energies for nine 1 bp mutants, as discussed in S2 Text. We then compare measured binding energies against predictions for 1, 2, and 3 bp mutants that were produced using either full-promoter energy matrices or operator-only energy matrices (see Figure 1). We find that operator-only energy matrices produce somewhat more accurate predictions than full-promoter energy matrices. We quantify this by noting the Pearson correlation coefficient (r) between the mean measured and predicted binding energies for each set of predictions, which indicates that the O1 operator-only matrix produces the most accurate predictions. The O2 operator-only matrix produces similarly accurate predictions. This shows us that operator-only energy matrices are a good option when it is feasible to infer the scaling factor from binding energy measurements.

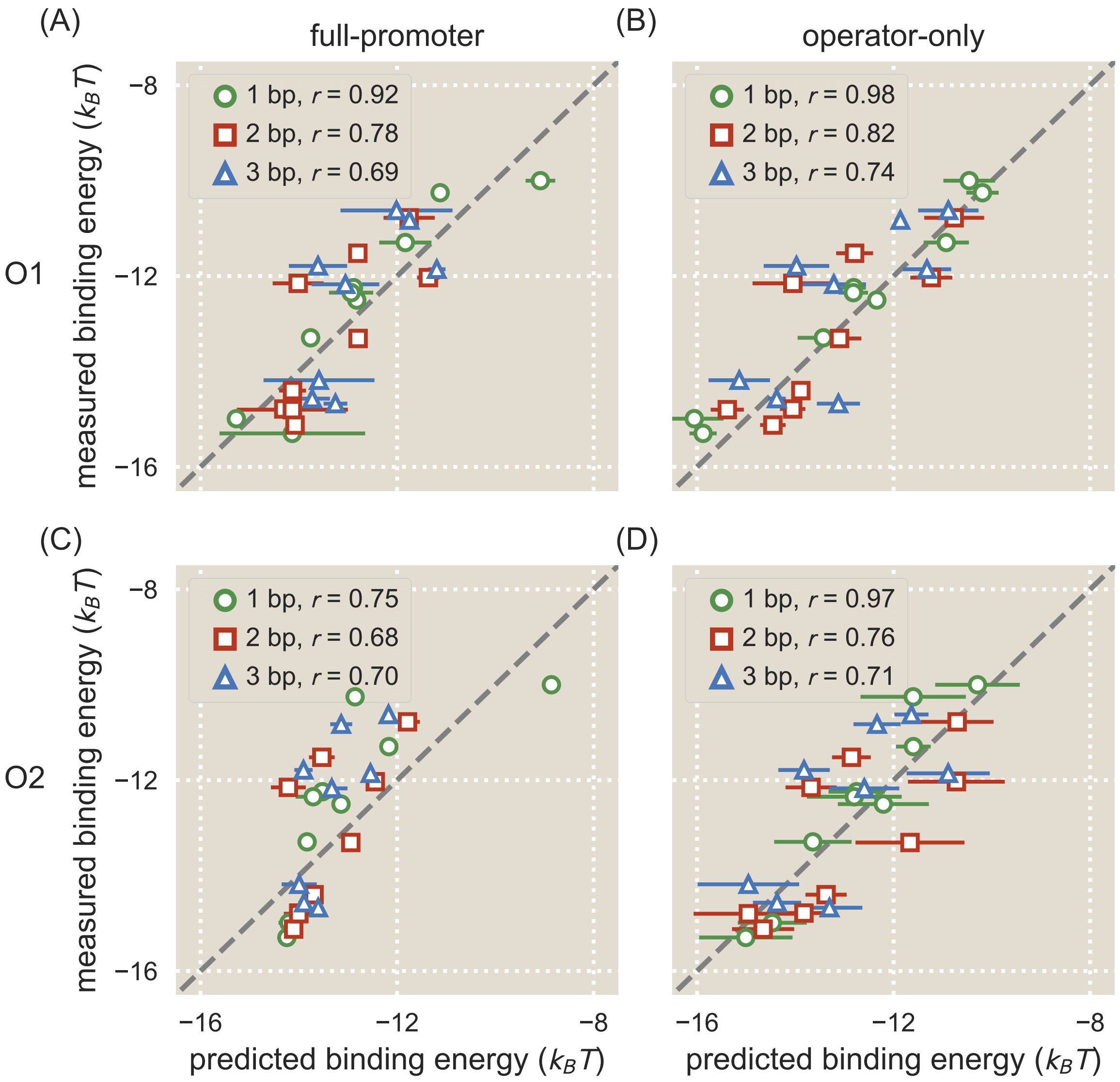

Figure 1. Mutating the operator alone can improve energy matrix accuracy. Binding energy measurements are plotted against energy matrix predictions from full-promoter (A, C) and operator-only (B, D) energy matrices using either O1 (A, B) or O2 (C, D) as a reference sequence. The Pearson correlation coefficient (r) is noted for each set of predictions. We see that the operator-only energy matrices produce more accurate predictions than the full-promoter energy matrices. Error bars represent the standard deviation of predictions from replicate energy matrices.

References
