# Supplementary material for: Mapping DNA sequence to transcription factor binding energy in vivo
Source: PLoS Comput Biol. 2019 Feb 4;15(2):e1006226. doi: 10.1371/journal.pcbi.1006226 (PMC6375646; doi:10.1371/journal.pcbi.1006226)
Supplement: S6 Text — (PDF) [file pcbi.1006226.s012.pdf]

Expressions for phenotypic parameters of induction responses

As discussed in greater detail in Ref. [1], the thermodynamic model we use to predict induction responses allows us to derive expressions for the phenotypic parameters of the induction response. Here we briefly list the expressions for the phenotypic parameters we address in the present work.

The leakiness of the induction curve is the minimum fold-change observed in the absence of ligand, given by

$$\begin{aligned} \text{leakiness} &= \text{fold-change}(c = 0) \\ &= \left(1 + \frac{1}{1 + e^{-\beta \Delta \varepsilon_{AI}}} \frac{2R}{N_{NS}} e^{-\beta \Delta \varepsilon_R}\right)^{-1}, \end{aligned} \tag{1}$$

where c is the concentration of inducer, n is the number of inducer binding sites on the repressor, and $\Delta\varepsilon_{AI}$ is the difference in free energy between the repressor's active and inactive states.

The saturation is the maximum fold change observed in the presence of saturating ligand,

$$\begin{aligned} \text{saturation} &= \text{fold-change}(c \to \infty) \\ &= \left(1 + \frac{1}{1 + e^{-\beta \Delta \varepsilon_{AI}} \left(\frac{K_A}{K_I}\right)^n} \frac{2R}{N_{NS}} e^{-\beta \Delta \varepsilon_R}\right)^{-1}, \end{aligned} \tag{2}$$

where K_A and K_I are the dissociation constants of the inducer and repressor when the repressor is in its active or inactive state, respectively.

Together, these two properties determine the dynamic range of a system's response, which is given by the difference

$$\text{dynamic range} = \text{saturation} - \text{leakiness}. \tag{3}$$

The full expression for dynamic range is then given by

$$\text{dynamic range} = \left(1 + \frac{1}{1 + e^{-\beta \Delta \varepsilon_{AI}} \left(\frac{K_A}{K_I}\right)^n} \frac{2R}{N_{NS}} e^{-\beta \Delta \varepsilon_R}\right)^{-1} - \left(1 + \frac{1}{1 + e^{-\beta \Delta \varepsilon_{AI}}} \frac{2R}{N_{NS}} e^{-\beta \Delta \varepsilon_R}\right)^{-1}. \tag{4}$$

The $[EC_{50}]$ of the induction response denotes the inducer concentration required to generate a system response halfway between its minimum and maximum value such that

$$\text{fold-change}(c = [EC_{50}]) = \frac{\text{leakiness} + \text{saturation}}{2}. \tag{5}$$

The full expression for the $[EC_{50}]$ is then given by

$$\frac{[EC_{50}]}{K_A} = \frac{\frac{K_A}{K_I} - 1}{\frac{K_A}{K_I} - \left(\frac{\left(1+\frac{2R}{N_{NS}} e^{-\beta\Delta\varepsilon_R}\right) + \left(\frac{K_A}{K_I}\right)^n \left(2e^{-\beta\Delta\varepsilon_{AI}} + \left(1+\frac{2R}{N_{NS}} e^{-\beta\Delta\varepsilon_R}\right)\right)}{2\left(1+\frac{2R}{N_{NS}} e^{-\beta\Delta\varepsilon_R}\right) + e^{-\beta\Delta\varepsilon_{AI}} + \left(\frac{K_A}{K_I}\right)^n e^{-\beta\Delta\varepsilon_{AI}}}\right)^{\frac{1}{n}}} - 1. \tag{6}$$

References

1. Razo-Mejia M, Barnes SL, Belliveau NM, Chure G, Einav T, Lewis M, et al. Tuning transcriptional regulation through signaling: A predictive theory of allosteric regulation. Cell Systems. 2018;In press.
